# Polymer-Grafted Nanoparticles as All-in-One Nanoplatforms

**DOI:** 10.1021/acspolymersau.6c00066

**Published:** 2026-04-29

**Authors:** Rongguan Yin, Xiaolei Hu, Hanshu Wu, Tong Liu, Michael R. Bockstaller, Krzysztof Matyjaszewski

**Affiliations:** † Department of Chemistry, 6612 Carnegie Mellon University, Pittsburgh, Pennsylvania 15213, United States; ‡ Department of Materials Science and Engineering, Carnegie Mellon University, Pittsburgh, Pennsylvania 15213, United States

**Keywords:** polymer-grafted nanoparticles, particle brushes, surface-functionalized nanoparticles, polymer nanohybrids, structure−property relationships, interparticle interactions, functional nanocomposites, hybrid nanomaterials

## Abstract

Polymer-grafted nanoparticles (PGNPs) represent a versatile class of hybrid nanomaterials, in which nanoparticle cores and tethered polymer coronas are integrated into structurally programmable building blocks. Rapid advances in nanoparticle surface functionalization and surface-initiated polymerization have enabled increasingly precise control over the nanoparticle core composition and brush architecture, greatly expanding the accessible structural and functional landscape of PGNPs. This review summarizes recent progress in the modular design and structural regulation of PGNPs, with an emphasis on nanoparticle platforms and associated surface functionalization strategies, polymer brush synthesis and architectural control, and the structure–property relationships that govern PGNP behavior. Emerging applications are further highlighted, including additive manufacturing, self-healing materials, membrane-based gas separations, and battery-related systems, where PGNPs provide unique opportunities to couple nanoscale interfacial design with macroscopic performance. Finally, future opportunities are discussed for extending PGNP concepts to increasingly complex, multifunctional, and application-oriented hybrid materials.

## Graphical abstract

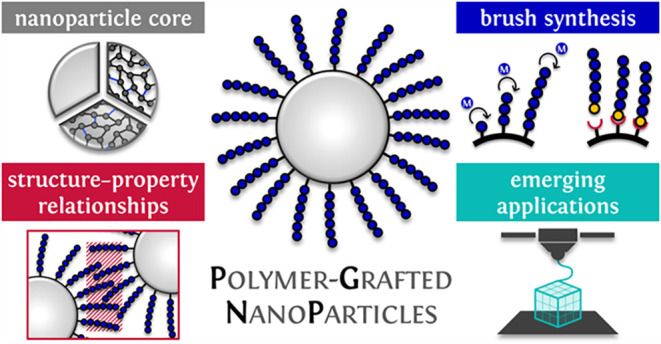

## 1 Introduction

Polymer-grafted nanoparticles (PGNPs), also referred to as organic/inorganic polymer nanohybrids, hairy or brush nanoparticles, and (nano)­particle brushes, constitute a versatile class of hybrid materials in which polymer chains are tethered to nanoparticle surfaces.^1–6^ The grafted brush layers transform inorganic or organic nanoparticle cores into architecturally defined building blocks with programmable interfacial characteristics. By decoupling interparticle interactions from the chemistry of the surrounding medium or matrix, PGNPs enable precise control over dispersion,^7–9^ assembly,^10–13^ and mechanical response.^14,15^ As a result, they have emerged not only as promising functional materials but also as powerful model systems for investigating confinement effects, brush-mediated repulsion, and nanoscale packing phenomena.^16–19^

Recent advances in nanoparticle surface functionalization and surface-initiated polymerization have greatly expanded the synthetic scope of PGNPs,^20–24^ enabling quantitative control over grafting density,^25^ chain uniformity,^26^ compositional gradients,^27^ and macromolecular topology.^28^ This level of synthetic precision has revealed how nanoscale parameters, such as initiator spatial distribution and local curvature,^29^ govern both the structure of the grafted corona and the emergent properties of concentrated brush assemblies and nanocomposites. As a result, PGNPs have evolved into a broadly adaptable platform for engineering hybrid nanomaterials with tailored optical, electrical, biomedical, and mechanical functionalities,^30–38^ while also serving as model systems for probing polymer physics under conditions of extreme geometric and compositional confinement.^39–43^

Against this backdrop, this review highlights progress in PGNP research, emphasizing advances that occurred during the past decade from the perspective of modular synthetic design and structure-enabled applications (Figure 1). We start by surveying nanoparticle platforms and surface functionalization strategies used to construct PGNPs from inorganic, organic, and hybrid cores. Methods for polymer brush synthesis and structural control are then discussed, including grafting strategies, controlled/living polymerization techniques, and the regulation of brush composition, architecture, dispersity, and chain length. Particular emphasis is placed on how these structural parameters govern brush conformation, interparticle interactions, collective behavior, and mechanical response. Emerging application areas, including advanced manufacturing, self-healing materials, membrane-based separations, and battery interfaces, are subsequently summarized, followed by an outlook on key challenges and future directions in the field.

**Figure 1:**
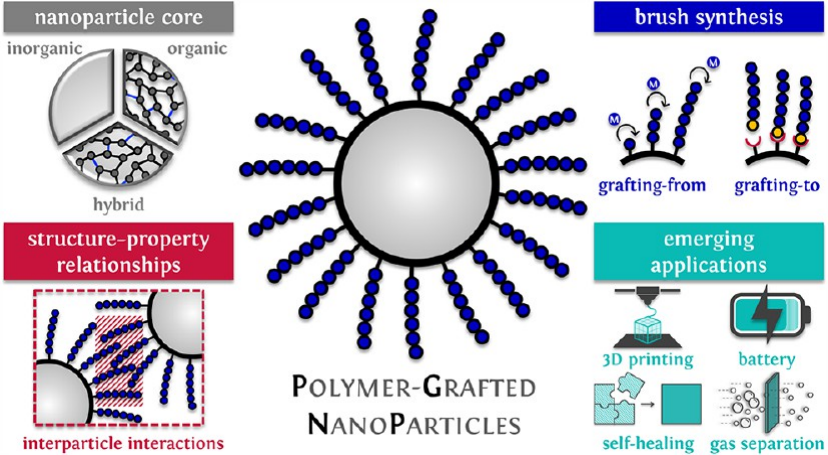
Modular design framework for PGNPs.

## 2 Nanoparticle Platforms and Surface Functionalization

### 2.1 Inorganic Cores

Inorganic nanoparticles possess high mechanical rigidity, excellent thermal and chemical stability, and offer versatile surface functionalization chemistry for polymer grafting.^44,45^ They often exhibit intrinsic functional properties, such as magnetism (e.g., Fe_3_O_4_),^46,47^ photocatalytic activity (e.g., ZnO),^48,49^ or photoluminescence as in the case of perovskite nanocrystals (e.g., CsPbBr_3_).^50^ A key advantage of PGNPs is the ability of the nanoparticle cores to retain these intrinsic properties while the surrounding polymer corona prevents aggregation and provides enhanced stability, compatibility, and processability.^1^ Achieving this synergy requires robust and chemically well-defined surface modification strategies that establish reliable anchoring sites for polymer growth.^51^

A diverse range of coupling chemistries has been developed to introduce initiators or reactive functionalities onto nanoparticle surfaces, enabling surface-initiated polymerization or surface tethering of chain-end-functionalized polymers in a well-regulated manner. The choice of anchoring reagents—including but not limited to silanes,^52–54^ catechols,^55,56^ carboxylates,^57,58^ phosphonates or phosphine oxides,^58–60^ and thiols or disulfides^61,62^ must be tailored to the specific surface chemistry of the nanoparticles. This ensures strong attachment, high grafting density, and reproducible brush growth.

Silane coupling agents are among the most widely used reagents for modifying silica nanoparticles (SiO_2_ NPs), as their alkoxy- or halide-silane groups efficiently react with surface silanol groups (Si–OH) to form robust Si–O–Si bonds.^63^ Their low cost, ease of handling, and compatibility with both laboratory-scale and industrial processes make silanes a practical and reliable choice for tailoring the interfacial chemistry of SiO_2_-based PGNPs.^64^ Moreover, silanes offer considerable versatility, as a broad library of commercially available derivatives enables the straightforward introduction of diverse surface functionalities. In many cases, mixtures of “active” and “dummy” silane initiators are employed to precisely regulate initiator density on SiO_2_ surfaces (Figure 2a).^65^ Active silanes bear initiating groups, whereas dummy silanes provide chemically similar but noninitiating anchoring moieties. The controlled dilution allows fine-tuning of the spacing between initiating sites, which in turn modulates interactions both within and between brush layers and thus contributes to the emergent morphological and mechanical properties of PGNP-based materials.

**Figure 2:**
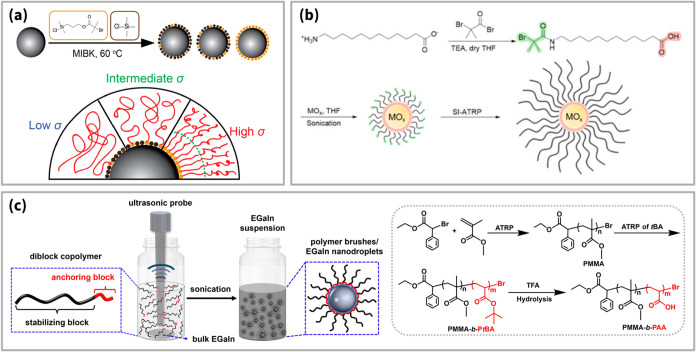
Representative strategies for constructing PGNPs using inorganic cores. (a) Surface modification of SiO_2_ NPs using mixed “active” and “dummy” silane initiators to regulate initiator spacing and grafting density (σ). Adapted with permission.^65^ Copyright 2020 American Chemical Society. (b) Synthesis of the fatty-acid-derived tetherable initiator BiBADA and its application in surface modification of metal oxide nanoparticles for subsequent polymer grafting. Adapted with permission.^57^ Copyright 2017 American Chemical Society. (c) Attachable diblock copolymers for the fabrication of polymer-tethered EGaIn nanodroplets via ultrasonication. Adapted with permission.^72^ Copyright 2020 American Chemical Society.

Metal oxide nanoparticles serve as robust, functionally rich cores in PGNPs and can be integrated with grafted polymer brushes to generate multifunctional hybrid materials. Inspired by the strong binding affinity of oleic acid toward metal oxide surfaces,^66^ a general surface modification strategy was developed based on the fatty-acid-derived tetherable initiator 12-(2-bromoisobutyramido)­dodecanoic acid (BiBADA) (Figure 2b).^57^ This BiBADA initiator enabled stable attachment of atom transfer radical polymerization (ATRP) initiating groups across a broad range of metal oxide nanoparticles, supporting a high grafting density of brush growth even on substrates that were previously difficult to functionalize. In contrast to phosphonate-based tetherable initiators, which are often synthetically demanding and limited in substrate scope, the BiBADA approach offers broad compatibility and simple processing, enabling uniform polymer-grafted metal oxide hybrid materials.

Eutectic gallium–indium (EGaIn) is a liquid metal alloy that can be dispersed within elastomeric matrices to produce soft composites with high thermal stability and electrical conductivity, making them attractive for applications in soft robotics and self-healing electronics.^67–70^ Surface functionalization with BiBADA enabled the formation of stable, solution-processable polymer-grafted EGaIn nanodroplets that exhibited exceptional dispersion stability, high loading capacity, and tunable mechanical and thermal properties.^71^ These hybrid nanostructures were directly cast into one-component soft materials, providing a versatile platform for flexible electronics that require uniform nanoscale liquid metal inclusions. An alternative strategy employed attachable diblock copolymers bearing a short poly­(acrylic acid) (PAA) anchoring block and a longer stabilizing block (Figure 2c).^72^ During ultrasonication, the PAA segments coordinated strongly with the native gallium oxide skin, enabling in situ formation of polymer-grafted EGaIn nanodroplets with high yield, narrow size distributions, and remarkable colloidal stability despite containing <1 wt % polymer.

### 2.2 Organic Cores

Compared with inorganic nanoparticle cores, organic nanoparticles (oNPs) offer greater structural and chemical tunability, as their size, composition, and internal rigidity can be systematically adjusted.^73–75^ This design flexibility allows oNPs to incorporate a wide range of small molecules, including fluorescent dyes,^76^ semiconducting units,^77^ and therapeutic agents.^78^ Their generally lower modulus can also provide complementary property enhancements such as rubber toughening. Moreover, because oNPs are constructed entirely from organic monomers, they retain chain-end functionality throughout the structure, eliminating the need for additional surface-modification steps that are typically required when polymers are grafted from inorganic substrates.

Lignin, an abundant and renewable biopolymer, can be converted to oNPs via acid-induced nanoprecipitation. The intrinsic hydroxyl groups of lignin acted as initiating sites for surface-initiated ring-opening polymerization (SI-ROP) of lactide, enabling grafting of poly­(lactic acid) (PLA) (Figure 3a).^79^ The resulting PLA-grafted lignin dispersed more uniformly in PLA matrices than in unmodified lignin, suppressing aggregation and phase separation during direct blending. At low lignin loadings (1–10 wt %), these PGNPs significantly enhanced UV-blocking and antioxidant performance while largely preserving the mechanical and barrier properties of PLA.

**Figure 3:**
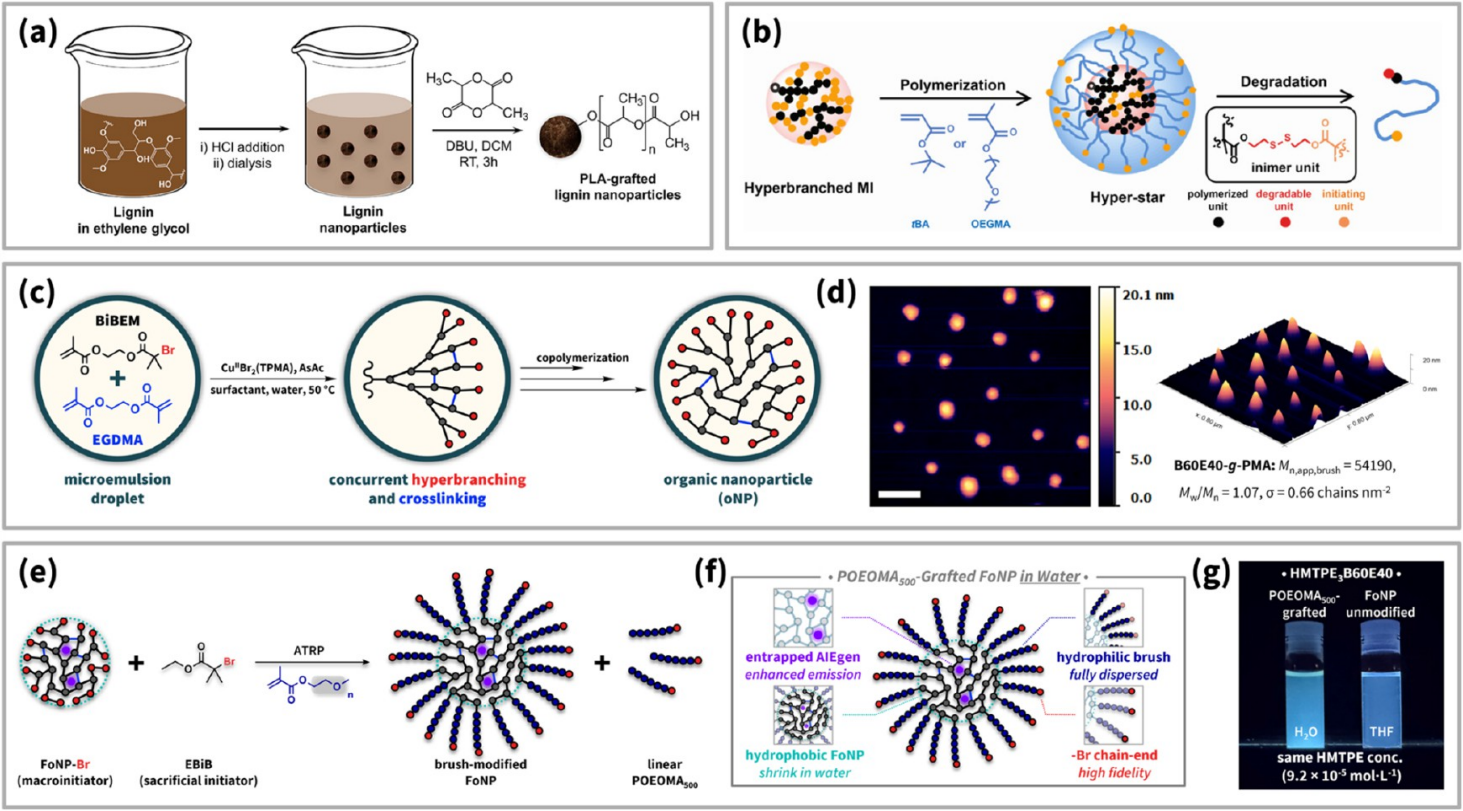
Representative strategies for constructing PGNPs using organic cores. (a) Formation of lignin nanoparticles via acid-induced nanoprecipitation followed by SI-ROP of lactide to produce PLA-grafted lignin nanoparticles. Reproduced from ref 79 with permission. Copyright 2022 American Chemical Society. (b) Chain extension of hyperbranched macroinitiators to generate hierarchical hyper-star architectures containing degradable inimer units. Adapted with permission.^82^ Copyright 2012 American Chemical Society. (c) Concurrent hyperbranching and cross-linking by microemulsion ATRP to produce structurally uniform oNPs with high chain-end fidelity. (d) AFM planar and 3D cross-sectional height images of poly­(methyl acrylate)-grafted oNPs with high grafting density. Scale bar: 160 nm. Adapted from ref 83 with permission. Copyright 2024 The Authors. Published by PNAS. (e) Surface-initiated atom transfer radical polymerization (SI-ATRP) from AIEgen-encapsulated FoNP-Br macroinitiator in the presence of a sacrificial initiator. (f) Schematic illustration of emission enhancement in brush-modified FoNPs. (g) FoNP grafted with poly­(oligo­(ethylene glycol) methyl ether methacrylate) (POEOMA_500_, average *M*_n_ = 500), exhibiting enhanced emission at low dye loading after grafting hydrophilic polymer brushes while remaining fully dispersed in water. Adapted from ref 85. Copyright 2025 The Authors. Published by the Royal Society of Chemistry under a CC BY-NC 3.0 license.

Microemulsions provide a suitable medium for oNP synthesis because they create nanoscale confined spaces in which polymer chains can grow into discrete nanoparticles at high monomer conversion.^80^ This compartmentalization restricts cross-linking reactions to individual droplets, enabling precise control over particle size, dispersity, and internal network structure.^81^ The resulting nanogels offered a well-defined platform for further chain extension, enabled by the high chain-end fidelity preserved in ATRP, and thus served as excellent cores for constructing hairy nanoparticles or organic PGNPs with tunable corona architectures. Beyond the formation of cross-linked nanogels, microemulsion ATRP can also regulate the growth of hyperbranched polymers within droplet boundaries.^82^ During inimer polymerization, in which each monomer carried an initiating site, microemulsions suppressed uncontrolled intermolecular coupling and enabled the formation of hyperbranched cores with an exceptionally high molecular weight and narrow dispersity. These uniform hyperbranched cores subsequently acted as multifunctional macroinitiators, allowing chain extension to generate hierarchical “hyper-star” architectures that could be designed to exhibit degradable or stimuli-responsive behavior (Figure 3b).

Building upon inimer chemistry in microemulsions, the design of oNPs has been further advanced by integrating concurrent hyperbranching and cross-linking to achieve precise control over core size and internal rigidity (Figure 3c,d).^83,84^ This strategy enabled the synthesis of structurally uniform oNPs with high chain-end fidelity, facilitating the efficient surface grafting of polymer brushes at high grafting densities. Extending this framework, aggregation-induced emission luminogen (AIEgen)-encapsulated fluorescent organic nanoparticles (FoNPs) were developed with intraparticle rigidity-regulated emission, highlighting the versatility of physical entrapment for constructing highly customizable fluorescent nanoparticle systems.^85^ Subsequent grafting of hydrophilic polymer brushes enhanced photoluminescence at low dye loading while maintaining full water dispersibility, thereby overcoming the traditional solubility limitations associated with AIEgens (Figure 3e–g).

In addition to physical encapsulation, fluorophores can be covalently incorporated as pendant groups, enabling backbone-stiffness-dependent photoluminescence.^86^ As the intraparticle rigidity increased, pyrene-containing FoNPs exhibited suppressed excimer formation, resulting in a progressive blue shift and dominance of monomer emission, whereas TPE-based FoNPs displayed enhanced AIE behavior. Solvent-dependent studies further revealed cooperative effects of the polarity and viscosity on the emission intensity. The retained chain-end fidelity afforded by ATRP also allowed FoNPs to function as multifunctional initiators and cross-linkers in UV-induced polymerization, yielding photoluminescent FoNP-oligo­(ethylene glycol) (OEG) hybrid gels.

### 2.3 Hybrid Cores

Organic–inorganic hybrid nanoparticles have emerged as attractive core candidates for PGNPs because they integrate the versatility of organic components with the structural robustness of inorganic materials. The inorganic domains typically provide mechanical robustness and thermal stability, while simultaneously imparting intrinsic functional properties. Meanwhile, the organic architecture offers chemically addressable surfaces that facilitate initiator immobilization and thus enable more stable brush grafting. Such systems therefore allow synergistic integration of core functionality with the tunable interparticle interactions provided by polymer coronas, expanding the design scope of PGNP-based nanocomposites.

Using silanes bearing ATRP-initiating groups eliminates the need for additional surface modification steps, yielding organosilica (oSiO_2_) nanoparticles with built-in ATRP capability.^87^ During synthesis, the initiator-containing silane precursors underwent self-condensation to form ultrasmall hybrid cores whose surface-bound α-bromoisobutyrate groups remained accessible for direct surface-initiated ATRP, enabling the growth of poly­(methyl methacrylate) (PMMA) brushes with grafting densities above 0.1 chain nm^–2^ (Figure 4a). The resulting brush particles exhibited a high organic content and excellent structural uniformity, establishing oSiO_2_ as a versatile platform for nanoscale SiO_2_-based hybrid materials. In a related study, these oSiO_2_ cores were used to grow dense polyacrylonitrile (PAN) brushes, producing uniform PGNPs that served as effective sacrificial templates for high-surface-area, nitrogen-enriched porous carbons (Figure 4b).^88^ Upon carbonization followed by SiO_2_ removal, the resulting nanoporous carbons exhibited exceptionally high-surface areas (>1200 m^2^·g^–1^) and uniform mesoporosity that directly reflected the dimensions of the oSiO_2_ templates. Related PAN-based grafting in silica-confined systems had been demonstrated even earlier in ordered mesoporous silicas, where SI-ATRP from concave cylindrical surfaces yielded thin PAN layers with tunable thickness and low dispersity while maintaining accessible porosity.^89^

**Figure 4:**
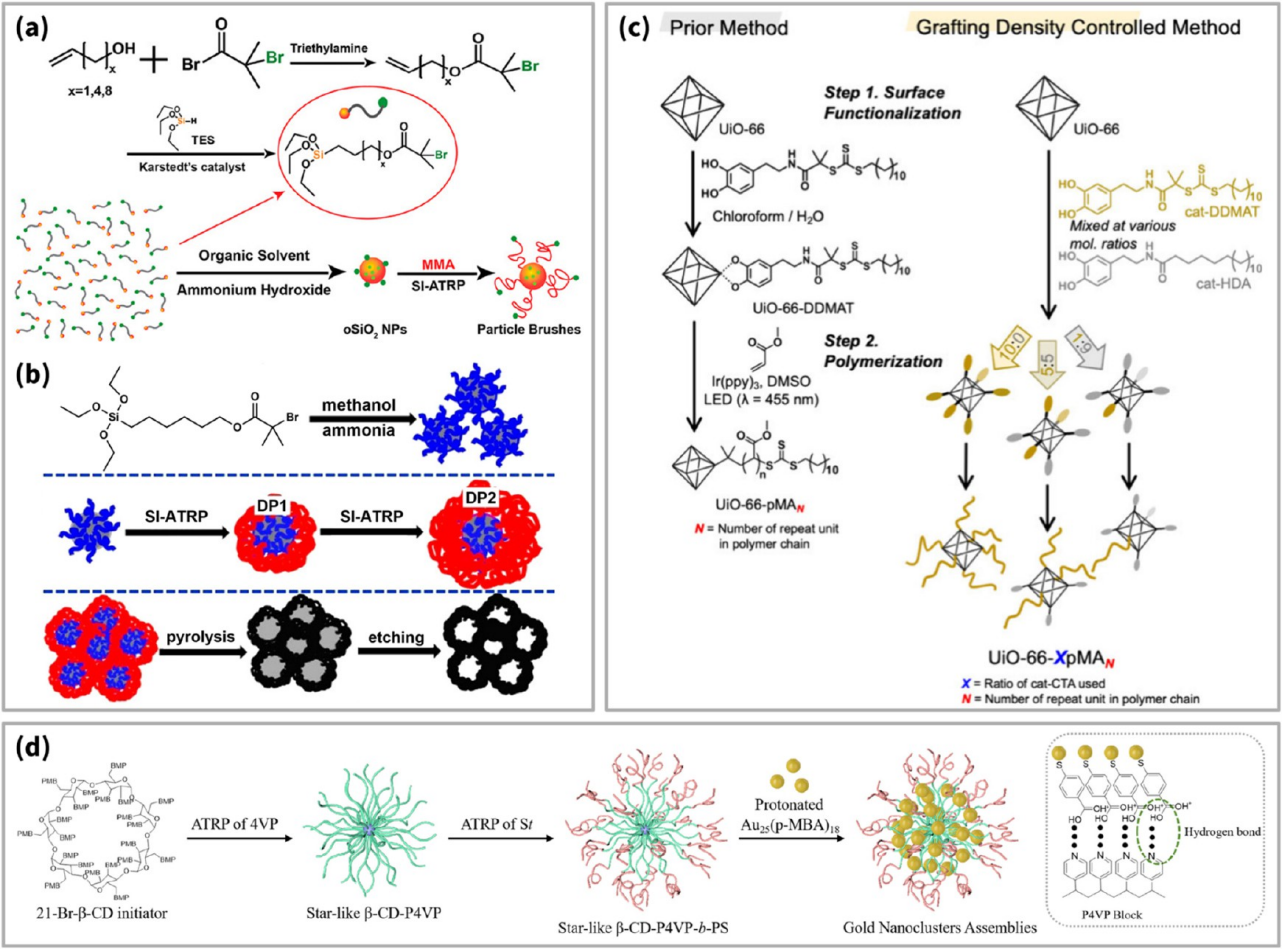
Representative strategies for constructing PGNPs using organic–inorganic hybrid cores. (a) Preparation of oSiO_2_ nanoparticles bearing ATRP initiators followed by SI-ATRP grafting PMMA polymer brushes. Adapted with permission.^87^ Copyright 2020 American Chemical Society. (b) Sequential SI-ATRP of PAN grafted from oSiO_2_ nanoparticles followed by pyrolysis and etching to generate nanoporous carbon materials. Adapted with permission.^88^ Copyright 2018 American Chemical Society. (c) Surface functionalization of MOF nanoparticles (UiO-66) with RAFT chain-transfer agents followed by photoinduced polymerization to produce polymer-grafted MOF hybrids, and regulation of polymer grafting density on MOF surfaces through mixed ratios of chain-transfer and block agents. Reproduced from ref 56 with permission. Copyright 2025 American Chemical Society. (d) Star-like diblock copolymers derived from β-cyclodextrin macroinitiators serving as nanoconfined templates for assembling Au nanoclusters, producing polymer-templated hybrid nanoparticles. Adapted with permission.^96^ Copyright 2026 American Chemical Society.

Metal–organic frameworks (MOFs), composed of metal nodes interconnected by organic linkers, represent another emerging class of hybrid cores for PGNPs.^90^ Their large surface areas and tunable pore environments enable multifunctional applications in catalysis, molecular separation, and therapeutic imaging. Beyond serving as passive porous supports, MOFs can also function as polymerization-active platforms or confined nanoreactors, enabling controlled radical polymerization either from functionalized framework surfaces or within their nanochannels.^91,92^ Grafting polymer brushes from MOF surfaces further enhanced the colloidal stability and interfacial compatibility, yielding polymer-grafted MOFs as functional nanocomposites with improved processability and performance. Surface modification could be achieved using a catechol-modified chain-transfer agents (CTAs) that coordinated to exposed metal nodes, enabling surface-initiated reversible addition–fragmentation chain-transfer (SI-RAFT) polymerization and subsequent self-assembly into densely packed free-standing monolayer films.^93^

The mechanical properties of these monolayers depend on the molecular weight and chemistry of the grafted polymer as well as the MOF particle size.^94^ Long, rubbery poly­(methyl acrylate) (PMA) brushes grafted from UiO-66 nanoparticles of intermediate size promoted interparticle entanglement and produced the most robust and flexible membranes. In a following study, PMA grafting density could be tuned by modifying the UiO-66 surface with mixed ratios of RAFT chain-transfer and blocking agents (which lacked chain-transfer functionality) (Figure 4c).^56^ Lower grafting density enabled longer polymer chains and improved particle ordering, whereas the mechanical robustness remained primarily governed by the polymer chain length.

Polymer-templated hybrid nanoparticles, in which Au nanoparticles nucleated and grew within the inner block of a star-diblock copolymer under nanoconfinement, were regarded as core–shell PGNPs featuring hybrid cores and a PMA brush corona.^95^ The outer PMA corona provided colloidal stabilization, while the chiral inner block transferred chirality to the confined Au nanoparticles, generating plasmonic assemblies that exhibited circular dichroism. In a related approach, star-diblock copolymer templates constructed by grafting poly­(4-vinylpyridine)-*block*-polystyrene from modified β-cyclodextrin (β-CD-*g*-P4VP-*b*-PS) were used to direct hierarchical nanoparticle assembly (Figure 4d).^96^ The P4VP inner block bound Au nanoclusters through hydrogen bonding interactions, while the outer PS block formed discrete surface patches under reduced solvent quality, enabling the step-growth-like assembly of hierarchical nanostructures.

## 3 Polymer Brush Synthesis and Structural Control

### 3.1 Grafting-from vs Grafting-to

Polymer brush tethering on nanoparticles is generally achieved through either grafting-from or grafting-to approaches, each associated with distinct advantages and limitations.^97,98^ The choice between these strategies depends on the nanoparticle surface chemistry and the targeted structural characteristics of PGNPs, and it strongly affects the attainable grafting density, compositional and orientational control, and even brush dispersity.^1^ Broadly, grafting-from is favored for generating dense brushes and enabling scalable chain growth, whereas grafting-to is particularly useful when preformed macromolecules with well-defined structures, precise sequence control, or delicate functionalities must be retained during surface attachment.

Grafting-from is the most widely used approach for preparing PGNPs and involves growing polymer chains directly from an initiator-functionalized nanoparticle surface (Figure 5a).^99^ This strategy enables high initiator densities because small initiator molecules are generally easier to immobilize than bulky preformed polymers, although its success depends on efficient surface functionalization and, in some cases, relatively complex reaction conditions. Grafting-from is readily compatible with a range of controlled polymerization techniques, providing access to dense brush regimes and convenient control over chain length through the monomer-to-initiator ratio and polymerization conditions.^100^ Representative methods include surface-initiated atom transfer radical polymerization (SI-ATRP),^23^ reversible addition–fragmentation chain-transfer (SI-RAFT) polymerization,^101^ nitroxide-mediated polymerization (SI-NMP),^102^ ring-opening polymerization (SI-ROP),^79^ and ring-opening metathesis polymerization (SI-ROMP).^103^ Nevertheless, accurate characterization of brush molecular weight is often less straightforward unless sacrificial initiators are employed or the grafted chains are cleaved from the surface for analysis.^104,105^ In addition, interparticle brush coupling and eventual macroscopic gelation can become significant at high monomer conversions, although this issue may be mitigated in heterogeneous media such as miniemulsion or microemulsion systems.^106^

**Figure 5:**
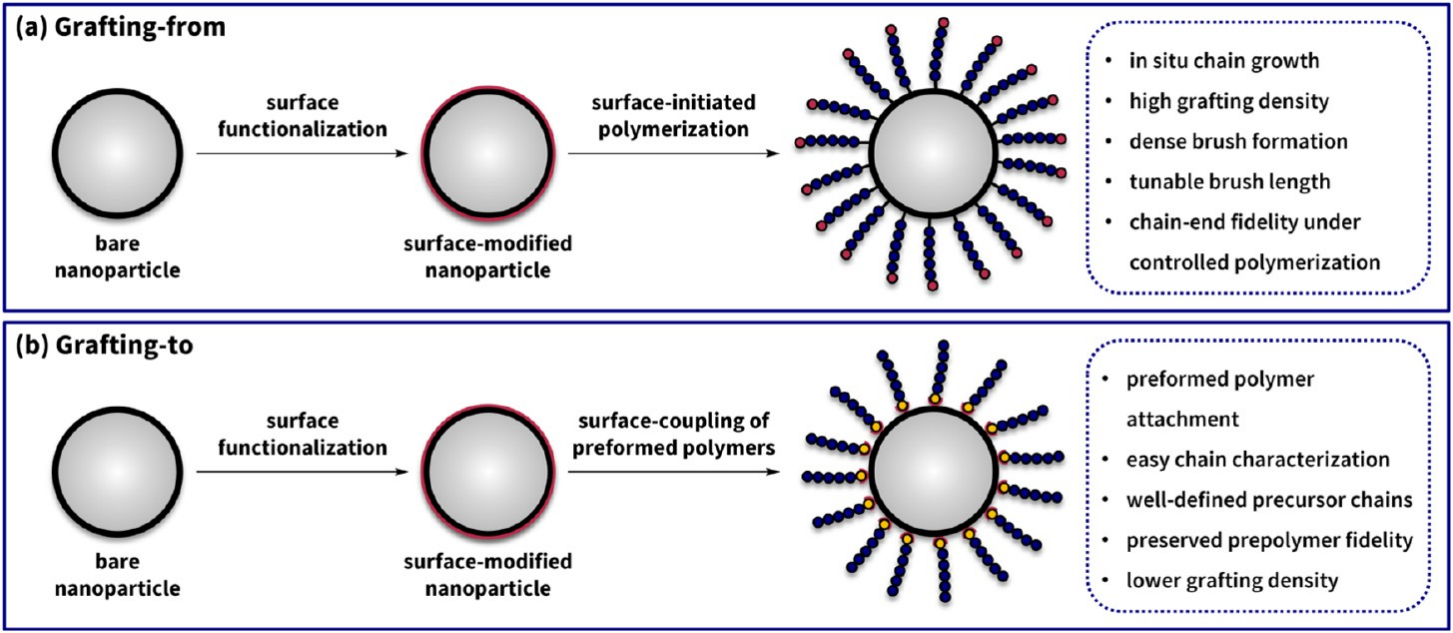
Schematic illustrations of the general strategies for PGNP synthesis by (a) grafting-from and (b) grafting-to. Depending on the nanoparticle surface chemistry, a separate surface-functionalization step may not always be required prior to polymer grafting.

Grafting-to involves the attachment of presynthesized polymer chains bearing reactive end groups to complementary anchoring sites on the nanoparticle surface (Figure 5b).^107^ Since the polymer chains are fully prepared before immobilization, key structural parameters, including molecular weight, dispersity, composition, sequence, and end-group fidelity, can be characterized in advance. As a result, this strategy is especially advantageous for discrete oligomers, block copolymers, functional polymers, and other systems in which precise chain identity is required.^72,108,109^ Its main limitation, however, lies in steric crowding during attachment, which restricts the achievable grafting density and often leads to lower-density brushes than those obtained by grafting-from.

### 3.2 Controlled/Living Polymerization Strategies

Surface-initiated reversible-deactivation radical polymerization (SI-RDRP) has become one of the most widely employed grafting-from approaches for synthesizing PGNPs.^110^ The two prominent examples are SI-ATRP and SI-RAFT polymerization.^20,100,111^ Despite their distinct mechanistic foundations (Figure 6a,b), both methods provide excellent control over the targeted molecular weight, low dispersity, and high grafting density. SI-ATRP proceeds through a dynamic redox equilibrium between dormant alkyl halides and propagating radicals mediated by a transition-metal catalyst (most commonly copper),^112,113^ whereas SI-RAFT relies on reversible chain-transfer equilibria directed by surface-anchored RAFT agents.^21,22^ Owing to their controlled/living characteristics, both methods also preserve chain-end fidelity, thereby enabling subsequent block extension and the construction of more complex brush architectures.

**Figure 6:**
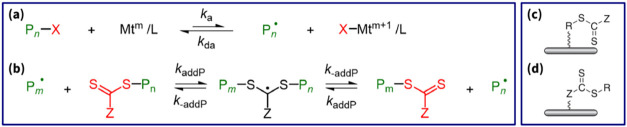
Simplified mechanisms of (a) ATRP and (b) RAFT polymerization. Adapted with permission.^110^ Copyright 2020 Elsevier B.V. Schematic illustrations of RAFT-agent surface immobilization through (c) R-group attachment and (d) Z-group attachment. Adapted with permission.^22^ Copyright 2022 Wiley-VCH GmbH.

For SI-RAFT in particular, the mode of surface anchoring is also a mechanistic consideration, because RAFT agents can be immobilized through either the R-group or the Z-group (Figure 6c,d), resulting in different polymer-growth pathways and chain-end locations.^22^ R-group-anchored SI-RAFT more closely corresponds to a true grafting-from strategy and is more commonly employed because the terminal chain-transfer functionality is retained at the outer chain end. In contrast, Z-group-anchored systems follow a distinct surface-mediated chain-transfer pathway and are therefore better considered separately. Although SI-ATRP and SI-RAFT do not offer fully identical monomer and functional-group compatibility, together they constitute complementary and versatile platforms for polymer growth from nanoparticle surfaces, allowing the synthetic method to be selected according to catalyst constraints, monomer scope, and targeted brush structure.

### 3.3 Oxygen-Tolerant Strategies

Oxygen limits the broad applicability of radical polymerization by quenching propagating radicals and oxidizing the catalytic species. Early oxygen-tolerant approaches primarily address residual oxygen through continuous redox regeneration, employing excess reducing agents or external stimuli such as photo-^114^ and electro- activation.^115^ However, full oxygen tolerance requires that the rate of oxygen consumption surpass oxygen diffusion into the reaction medium, thereby enabling polymerization under open-air conditions.^116^ Highlighted strategies include enzymatic oxygen depletion via glucose oxidase,^117,118^ photocatalyst-enabled redox cycling,^119–123^ and alkylborane autoxidation that concurrently consumes oxygen and regenerates activators.^124^ These advances eliminate the need for rigorous deoxygenation and significantly enhance the practicality of radical polymerization.

The primary limitation of applying photoredox cocatalysis to open-air PGNP synthesis arises from the concomitant generation of new radicals, which can initiate additional polymer chains during polymerization.^124^ Even a small fraction of unattached free polymer chains constitutes a significant impurity in bulk PGNP film fabrication, potentially compromising the resulting material properties.^125^ When nanoparticles are insufficiently large (and thus possess limited mass), the separation of these free chains becomes challenging. Although substantial progress has been achieved in oxygen-tolerant grafting from flat substrates where excess free chains can be readily removed by simple washing (Figure 7),^126–133^ fully oxygen-tolerant PGNP synthesis with high target purity suitable for large-scale and efficient fabrication remains unresolved. A practical compromise may involve conducting photocatalyst-mediated grafting from relatively large nanoparticles (*d*_core_ > 100 nm), for which newly formed free chains can be effectively removed by high-speed centrifugation.

**Figure 7:**
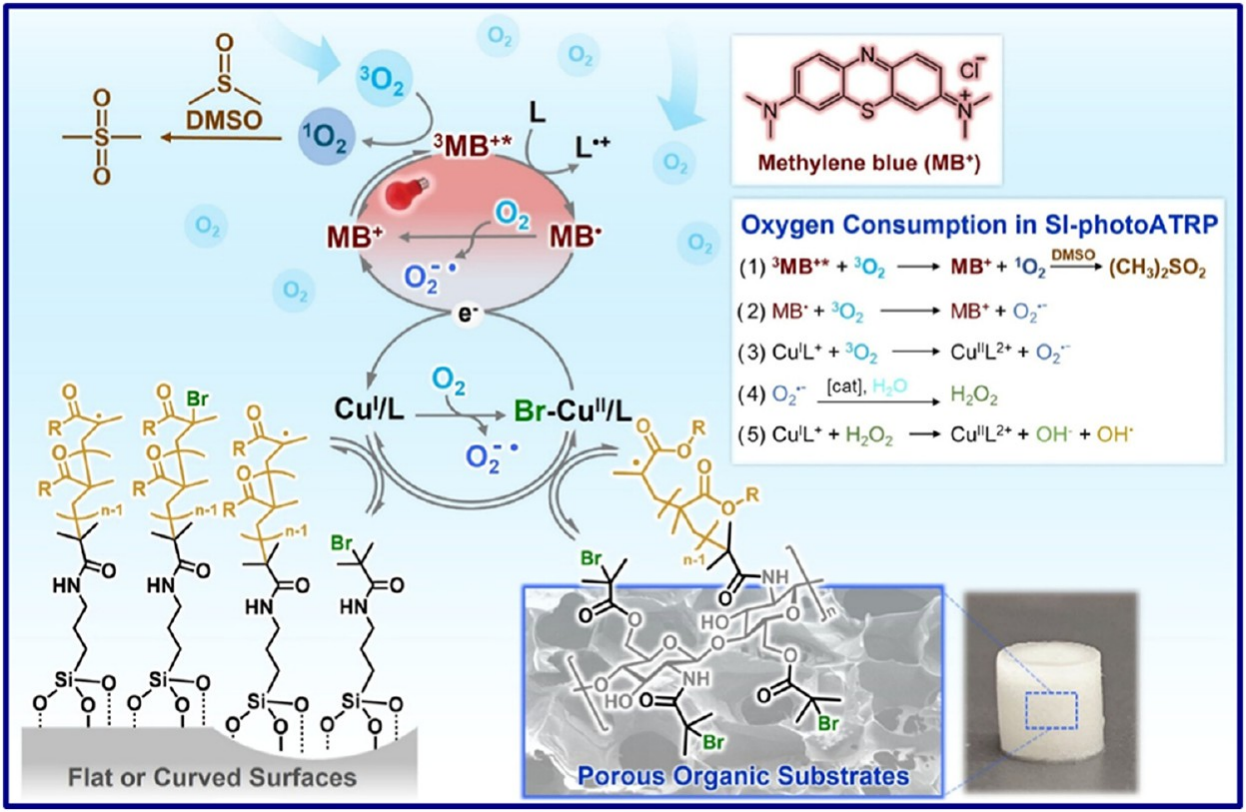
Schematic illustration of surface-initiated photoATRP enabling brush growth from initiator-functionalized flat, curved, or porous substrates mediated by methylene blue (MB^+^) under red-light irradiation in open air. Adapted from ref 131 Copyright 2025 The Authors. Published by American Chemical Society under a CC BY 4.0 license.

### 3.4 Brush Composition and Architectural Design

Compositional control enables systematic tuning of interfacial properties of PGNPs, including wettability, assembly behavior, and compatibility with surrounding matrices.^5^ Classical copolymer architectures can also be implemented in polymer brushes grafted from nanoparticle surfaces, including block, statistical, and gradient copolymers. Block copolymer brushes exploit the preserved chain-end functionality afforded by controlled polymerization techniques, enabling sequential chain extension to produce either uniform brushes or intentionally nonuniform architectures with distinct compositional segments.^65,134,135^ Statistical copolymer brushes are typically synthesized by copolymerizing multiple monomers in a single step, where differences in the comonomer reactivity ratios and initial feed compositions determine the final chain composition. In some cases, a nonequimolar monomer feed combined with termination at low overall conversion was employed to better regulate the statistical incorporation of comonomers along the brush chains.^136,137^

Spontaneous gradient copolymers in the unattached linear form typically require very high conversions of comonomers with significantly different reactivity ratios.^138^ Compared with their linear analogues, gradient copolymers grafted from nanoparticles exhibited an additional structural parameter, namely the chain growth orientation, where the radial composition from the nanoparticle surface toward the chain termini became a defining characteristic.^139^ However, in homogeneous PGNP syntheses, monomer conversions are limited to below ∼20% to prevent interparticle brush coupling, which would otherwise lead to macroscopic gelation. To address this limitation, the monomer with the higher reactivity ratio (methyl methacrylate, *r*_MMA_ = 2.07) was gradually fed into the less reactive monomer (*n*-butyl acrylate, *r*_BA_ = 0.35) (Figure 8a).^27^ By controlling the initial concentration of BA and the feeding rate of MMA while maintaining low overall conversions, SiO_2_-*g*-PBA-*grad*-PMMA particle brushes with a softer, PBA-rich inner segment were obtained together with tunable compositional gradients along the chains. Alternatively, spontaneous gradient copolymer brushes were synthesized in miniemulsion systems, where polymerization occurred within confined micellar compartments that suppressed interparticle brush coupling and prevented macroscopic gelation (Figure 8b–d).^106^ The resulting SiO_2_-*g*-PMMA-*grad*-PBA developed progressively along the chain growth direction with nearly complete conversion, yielding polymer brushes with low dispersity, high grafting density, and well-defined compositional gradients.^139^

**Figure 8:**
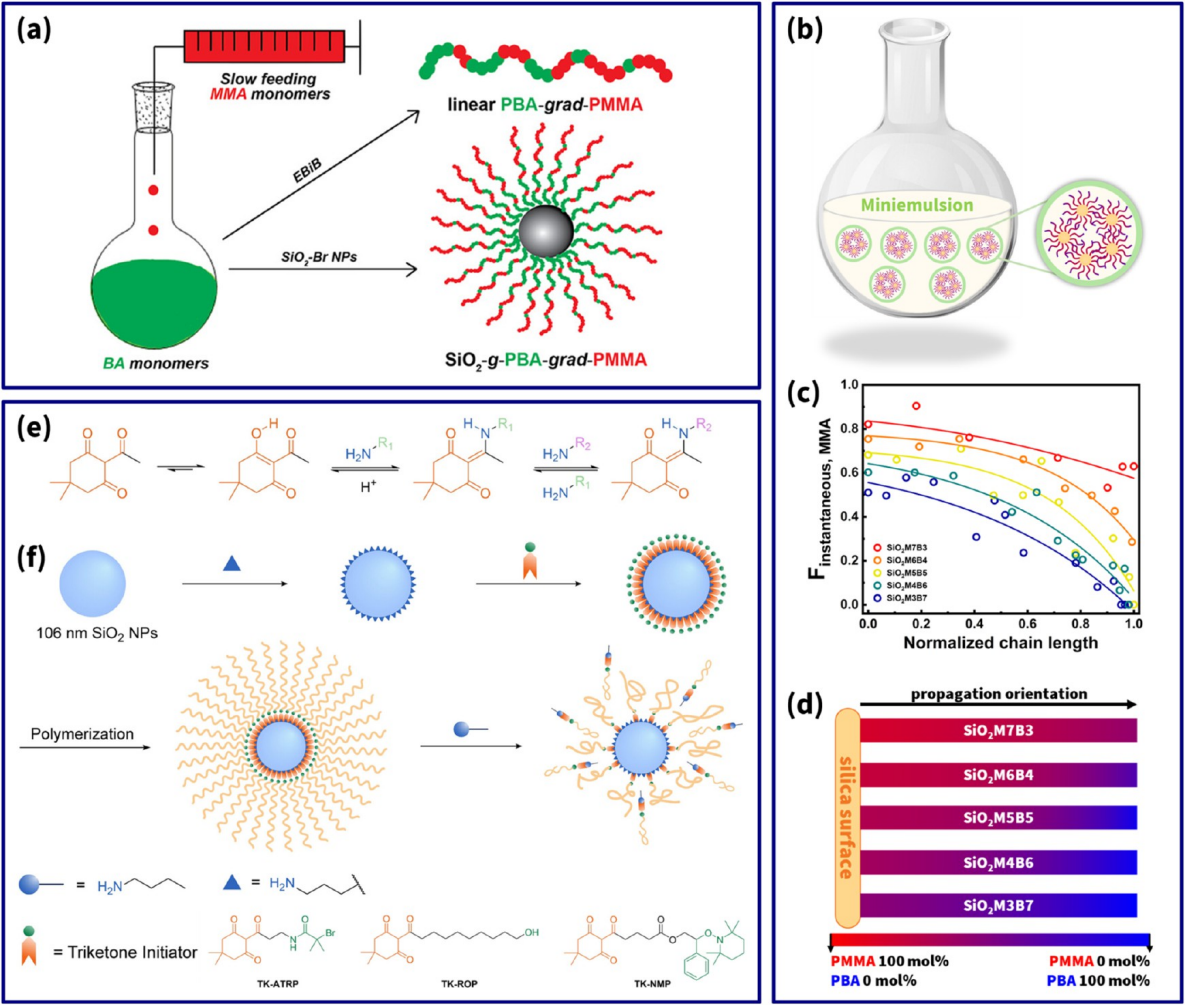
Representative strategies for constructing PGNPs with controlled brush composition and architecture. (a) Synthesis of linear PBA-*grad*-PMMA and SiO_2_-*g*-PBA-*grad*-PMMA PGNPs featuring a softer PBA-rich inner segment. Adapted with permission.^27^ Copyright 2019 American Chemical Society. (b) Schematic illustration of PGNP synthesis in miniemulsion, where compartmentalization prevents macroscopic gelation at high comonomer conversions. (c) Instantaneous incorporations of MMA into the copolymer chains. The fitted curves serve as guides to the eye. (d) Illustration of the gradient compositional change along polymer chains following the propagation orientation of copolymer grafting from the SiO_2_ NP surface using RGB color coordinates (red, green, blue). For example, 100 mol % PMMA corresponds to (255, 0, 0), whereas 100 mol % PBA corresponds to (0, 0, 255). 50 mol % PMMA and 50 mol % PBA correspond to (127, 0, 127). Adapted from ref 139. Copyright 2023 The Authors. Published by American Chemical Society under a CC BY 4.0 license. (e) Reaction scheme between diketone and amines. (f) Schematic illustration of the introduction of triketone initiators and subsequent polymerization from SiO_2_ NP surfaces via the grafting-from strategy. Adapted with permission.^140^ Copyright 2024 Wiley-VCH GmbH.

Incorporating dynamic or reversible bonding motifs at particle interfaces or within brush-based architectures enables selective cleavage, regrowth, or reconfiguration of polymer-grafted systems.^140,141^ Linkages such as diketoenamine provided tunable control over grafting density and chain length,^142^ thereby expanding architectural design beyond conventional irreversible grafting-from strategies (Figure 8e,f). Furthermore, sequential regrafting via orthogonal polymerization techniques permitted the construction of mixed and multimodal brush architectures on a single surface, offering a versatile platform for reconfigurable PGNP systems.^140^

### 3.5 Dispersity and Chain Length

Polymer dispersity (*Đ*), defined as the ratio of weight-average to number-average molecular weight (*M*_w_/*M*_n_), reflects the breadth of the molecular weight distribution in a polymer sample. The dispersity of grafted brushes plays a critical role in governing interparticle interactions and the assembly behavior of the PGNP materials. A comprehensive overview of dispersity effects in grafted brushes has been discussed previously.^143^ Although uniform polymers have traditionally been regarded as an important objective in polymer science,^144–146^ increasing evidence suggests that different material properties exhibit distinct dependencies on *Đ*.^147^ As a result, deliberate tuning of *Đ* enables its use as a design parameter for tailoring the PGNP assembly and accessing new nanocomposite materials.

The *Đ* of brushes grown from nanoparticles by ATRP can be tuned by varying the Cu catalyst concentration ([Cu^II^Br_2_]_0_).^148^ Above a threshold (∼10 ppm relative to the monomer), efficient initiation yielded densely grafted brushes with low *Đ* and isotropic SiO_2_ NP distributions. However, lower [Cu^II^Br_2_]_0_ resulted in reduced grafting density and higher *Đ* due to slower deactivation, leading to patchy nanoparticle surfaces that self-assembled into anisotropic string-like structures (Figure 9a,b).

**Figure 9:**
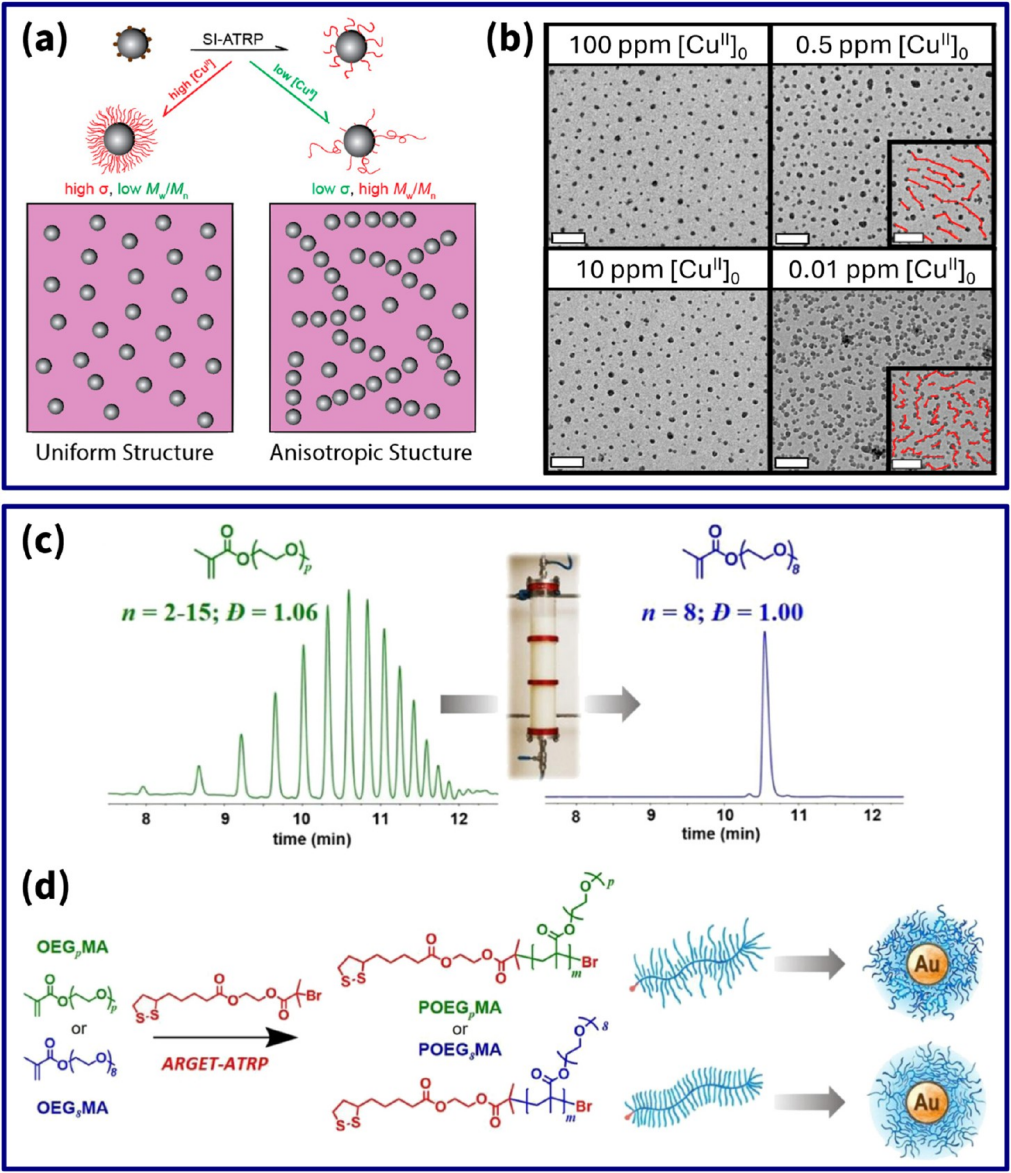
Representative strategies for constructing PGNPs with controlled dispersity. (a) Schematic illustration of PGNP synthesis via SI-ATRP, and (b) TEM images of monolayer films of SiO_2_-*g*-PMMA prepared using different initial Cu catalyst concentrations. Red lines in the insets highlight string-like structures. Scale bar: 100 nm. Adapted with permission.^148^ Copyright 2019 American Chemical Society. (c) UPLC traces showing the *M*_n_ distribution of OEG_*p*_MA containing oligomers from *n* = 2–15 and discrete OEG_8_MA (*n* = 8) obtained after chromatographic purification. Adapted with permission.^151^ Copyright 2024 American Chemical Society. (d) ARGET-ATRP from disulfide-based initiators yields structurally heterogeneous POEG_*p*_MA and discrete POEG_8_MA, which are subsequently attached to Au NPs via the grafting-to method. Adapted from ref 62. Copyright 2025 The Authors. Published by American Chemical Society under a CC BY 4.0 license.

The concentration of nanoparticle macroinitiator ([SiO_2_–Br]_0_) may also influence the *Đ* of grafted brush layers, although this typically occurs at the expense of grafting density.^149^ For PS-grafted SiO_2_ NPs, decreasing [SiO_2_–Br]_0_ lowered the initiation efficiency and increased the relative contribution of termination reactions, leading to higher *Đ* values and changes in PGNP morphology. In contrast, PMMA-grafted analogues maintained relatively low *Đ* over a similar [SiO_2_–Br]_0_ range, attributed to the localization of initiating sites on nanoparticle surfaces that reduced the probability of radical coupling between growing chains. This concept enabled the preparation of ultrahigh molecular weight SiO_2_-*g*-PMMA particle brushes (*M*_n_ > 1 MDa) with relatively low *Đ* even under low monomer conversion.^150^

The dispersity of pendant side chains within the brush layers of PGNPs has emerged as an under-appreciated design parameter. This was examined by comparing gold nanoparticles grafted with polydisperse POEG_*p*_MA (derived from commercial poly­[oligo­(ethylene glycol) methacrylate] macromonomers) and structurally uniform POEG_8_MA (as the discrete oligomer isolated via flash chromatography) (Figure 9c,d).^151^ Homogeneous POEG_8_MA brushes exhibited enhanced colloidal stability across a broad temperature range, whereas the presence of longer OEG segments in polydisperse brushes acted as epitopes, thereby promoting stronger anti-PEG antibody recognition.^62^

## 4 Structure–Property Relationships in PGNPs

### 4.1 Grafting Density and Brush Regimes

Grafting density (σ, chains nm^–2^) defines the number of polymer chains grafted per unit surface area.^25^ In PGNPs, it governs the extent of lateral crowding at the nanoparticle surface and, therefore, strongly influences brush conformation and interpenetration. At the same time, grafting density is closely coupled with other structural parameters, particularly chain length and *Đ* of the grafted chains, such that its effects are often reflected in complex structure–property relationships. As grafting density increases, neighboring chains become progressively more confined, driving the transition from mushroom-like conformations to semidilute particle brush (SDPB) and ultimately concentrated particle brush (CPB) regimes (Figure 10a).^1,5,13,152^ These conformational changes, in turn, strongly affect PGNP dispersion, assembly, rheological behavior, and mechanical reinforcement. Accordingly, precise regulation and reliable quantification of grafting density are essential for establishing design rules in PGNP systems and understanding how nanoscale interfacial architecture governs macroscopic material performance.

**Figure 10:**
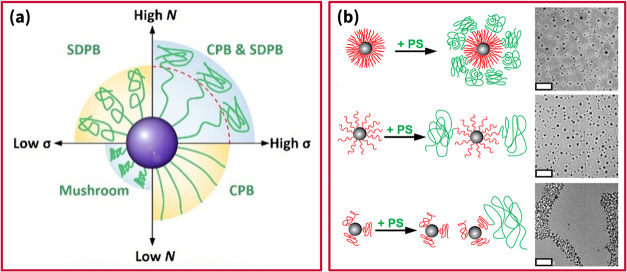
Grafting density effects on PGNPs. (a) Schematic illustration of the evolution of brush conformations with grafting density and chain length. Adapted with permission.^13^ Copyright 2026 American Chemical Society. (b) Schematic illustration and representative TEM images of bimodal SiO_2_-*g*-PMMA-*b*-PS particle brushes derived from densely, moderately, and sparsely grafted chains. Scale bars: 100 nm. Adapted with permission.^65^ Copyright 2020 American Chemical Society.

In polymer-grafted MOFs, grafting density has been shown to strongly influence brush morphology and interparticle packing.^56^ Specifically, lowering the grafting density reduced the increase in brush height with chain length and promoted more ordered particle arrangements. Sparsely grafted MOFs could still assemble into free-standing monolayers, meaning that low grafting densities maintained sufficient interparticle cohesion when the grafted chains were long enough. Mechanically, however, the effect of grafting density appeared less dominant than that of chain length, with no strong correlation between grafting density and monolayer toughness.

Bimodal block copolymer particle brushes with tunable assembly were synthesized using SiO_2_ NPs with different initiator densities, in which a primary PMMA graft layer was chain-extended with PS as the second block.^65^ The extent of chain extension and the resulting dispersity were further regulated by the Cu catalyst concentration during the second-step ATRP. At a low extension efficiency (∼7%), the resulting phase-separated morphology was attributed to segregation between PMMA-grafted particles and PMMA-*b*-PS-modified particle brushes. As the fraction of PMMA-*b*-PS chains increased, the microstructure became progressively more uniform and evolved into anisotropic string-like assemblies. Moreover, when the initial PMMA block was prepared at high, medium, and low grafting densities, the corresponding chain-extended particle brushes exhibited distinct morphologies, including relatively uniform structures with partially string-like features, connected string networks, and continuous cluster networks, respectively (Figure 10b). These results demonstrated that grafting density provided an effective handle for directing the self-assembly of bimodal block copolymer particle brushes and offered a route to hierarchically ordered quasi-one-component materials.

### 4.2 Curvature Effects

Early discussion of nanoparticle curvature effects emerged from a theoretical framework, showing that curvature governed the behavior of PGNPs in chemically identical polymer matrices.^153^ Increasing curvature (corresponding to a smaller particle size) reduced brush crowding and promoted matrix-chain penetration into the grafted layer, whereas lower curvature, higher grafting density, and larger matrix-to-graft molecular weight ratios favored brush dewetting. These curvature-dependent changes in brush–matrix interpenetration further regulated interparticle interactions. Greater wetting led to weaker attraction or more repulsive interactions, whereas dewetting produced more attractive potentials and a greater tendency toward aggregation.

The nanoparticle curvature also influences polymer brush growth during SI-RDRP. As grafted chains grew from the surface, increasing confinement prevented a fraction of dormant chain ends from being reactivated, leaving them kinetically trapped as hindered dormant chains rather than truly terminated species (Figure 11a).^154^ This confinement-induced heterogeneity led to broader and even bimodal chain length distributions within the grafted layer, although such heterogeneity might be obscured in conventional molar-mass analyses. Importantly, the effect became more pronounced as particle radius increased and surface curvature decreased since flatter surfaces exacerbated chain crowding and reduced control over brush growth relative to highly curved nanoparticles.

**Figure 11:**
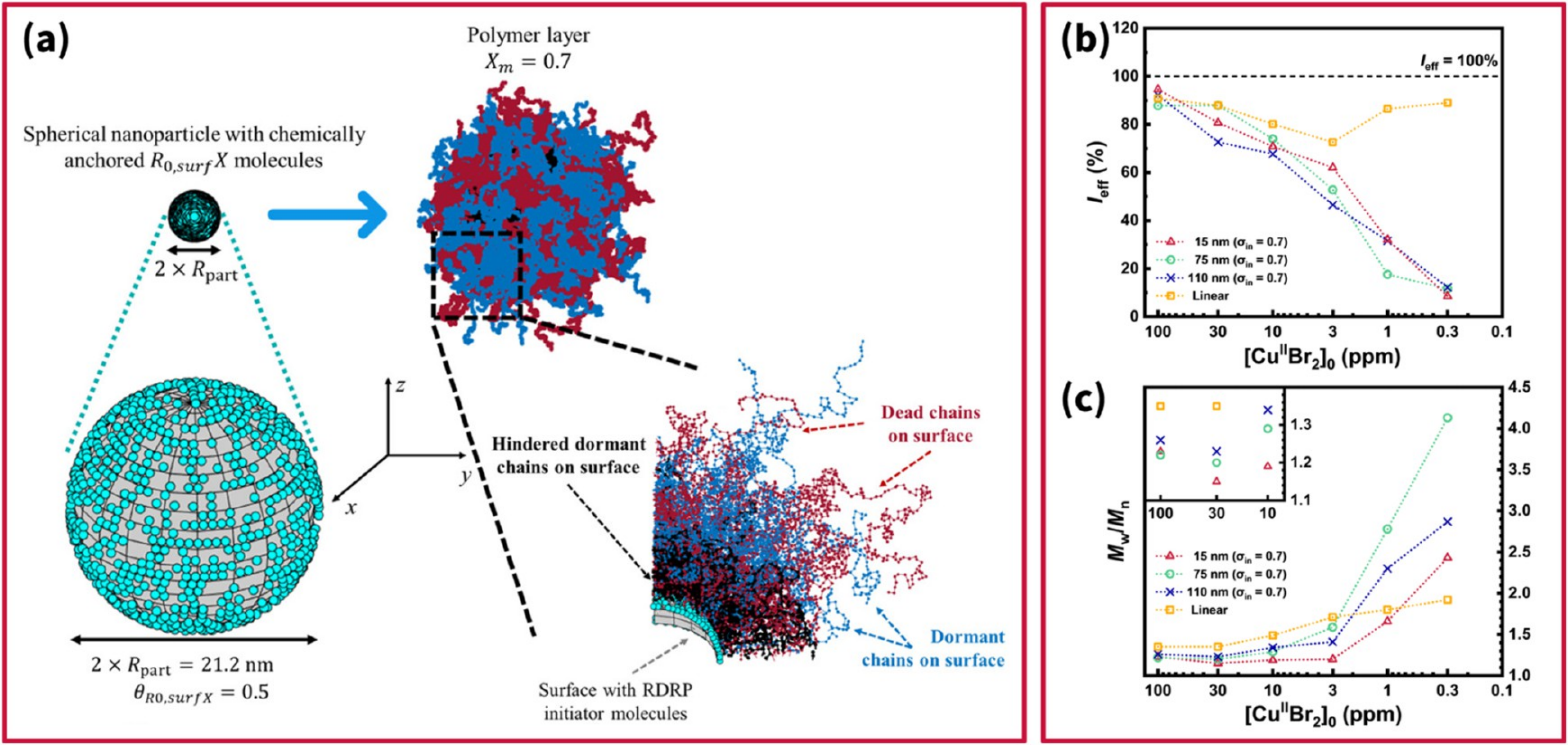
Curvature effects on PGNPs. (a) Schematic representation of a spherical nanoparticle model used to examine curvature-dependent brush growth during SI-RDRP, with particle radius *R*_part_ = 10.6 nm, initiator density θ_R0,surfX_ = 0.5, monomer conversion *X*_m_ = 0.7, and a targeted degree of polymerization of 100. Surface-grafted chains are classified as dormant chains (blue), dead chains (red), and hindered dormant chains (black). Adapted with permission.^154^ Copyright 2021 American Chemical Society. (b) Initiation efficiency (*I*_eff_) and (c) molecular weight distribution (*M*_w_/*M*_n_) as a function of initial Cu catalyst concentrations ([Cu^II^Br_2_]_0_), showing experimental comparisons among SiO_2_-*g*-PMMA with different surface curvatures and PMMA free polymers under analogous (SI-)­ATRP conditions. Adapted from ref 29. Copyright 2026 The Authors. Published by American Chemical Society under a CC BY 4.0 license.

Building on the computational picture, the subsequent experimental study showed that initiator density, catalyst concentration, and nanoparticle curvature collectively determined the uniformity of brush growth during SI-ATRP from spherical SiO_2_ NPs.^29^ Higher initiator density and lower surface curvature increased steric crowding among growing chains, leading to a reduced initiation efficiency and higher *Đ*. Comparison with unattached PMMA free polymers further supported the existence of permanently buried initiation sites unique to surface-grafted systems (Figure 11b,c), thereby providing experimental support for the hindered dormant chains predicted by simulation.

### 4.3 Interparticle Brush Interactions

PGNPs can self-organize into colloidal polymers, superlattices, and other structurally diverse assemblies through tunable brush-mediated interparticle interactions.^155^ Such a collective organization is governed not only by electrostatic, hydrogen-bonding, halogen-bonding, and other recognition-driven pathways,^13^ but also by brush conformation, interpenetration, and enthalpic/entropic effects under soft confinement.^10^ As a typical example, oppositely charged polyelectrolyte-grafted nanoparticles were shown to form substrate-supported colloidal molecules with tunable coordination numbers and geometries through electrostatic complexation between complementary brushes (Figure 12a–c).^156^ The pH, ionic strength, and charge matching jointly regulated the balance among electrostatic attraction and interparticle repulsion, enabling the controlled evolution of AB_*x*_ structures and their spatial arrangement on substrates.

**Figure 12:**
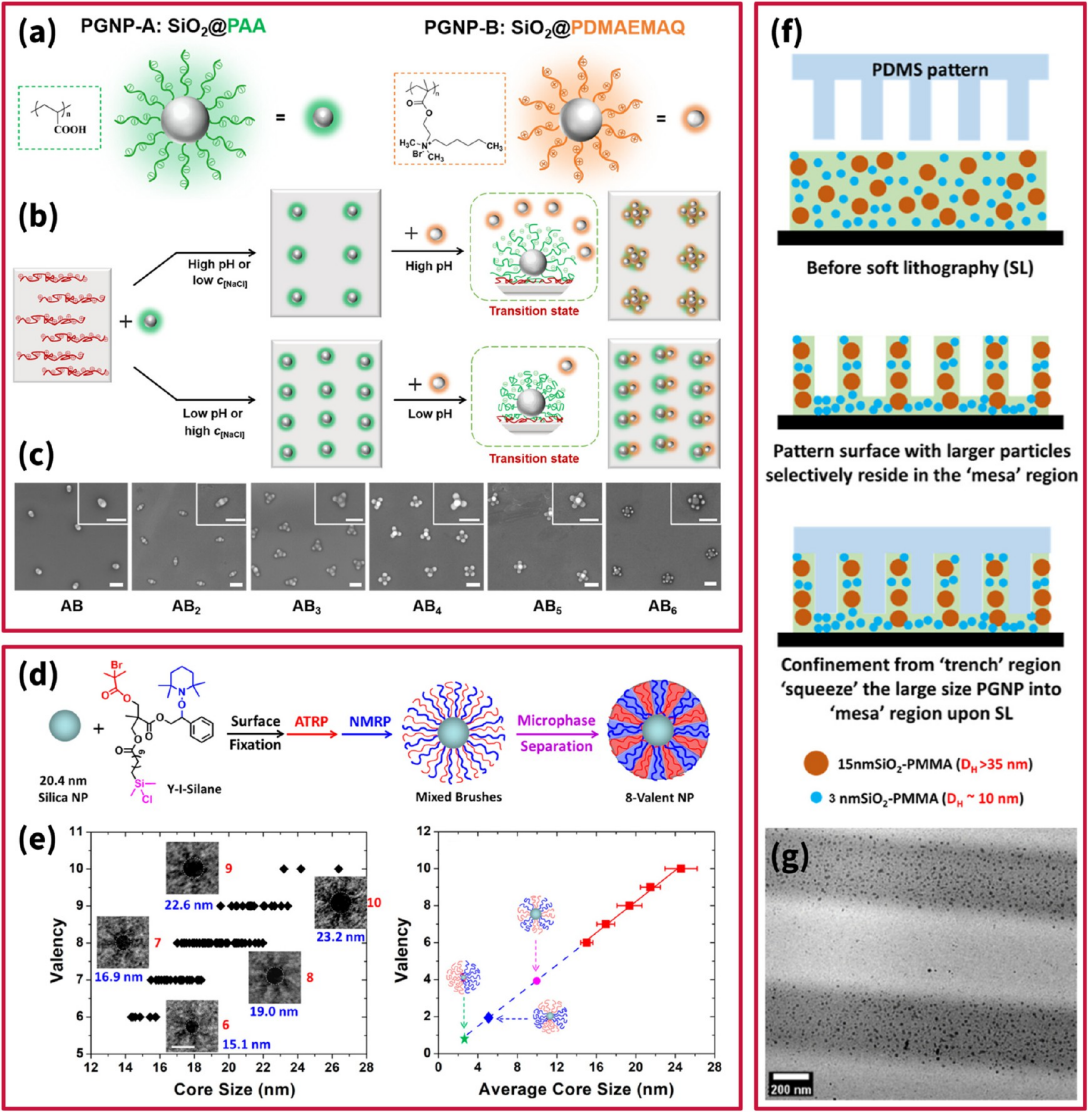
Interparticle brush interactions of PGNPs. (a) Schematic illustration of oppositely charged polyelectrolyte-grafted nanoparticles and (b) their self-assembly into substrate-supported colloidal macromolecules. (c) SEM images of representative AB_*x*_ structures obtained from different PGNP building blocks. Adapted with permission.^156^ Copyright 2024 American Chemical Society. (d) Synthetic route to binary mixed homopolymer brush (P*t*BA/PS)-grafted SiO_2_ NPs and the formation of multivalent patchy nanoparticles through lateral microphase separation. (e) Valency plotted as a function of core size (left) or average core size (right). Scale bar: 25 nm. The star, diamond, and circle denote mono-, di-, and tetravalent NPs obtained by extrapolation, respectively. Adapted with permission.^158^ Copyright 2021 American Chemical Society. (f) Schematic illustration of the selective partitioning of SiO_2_-*g*-PMMA blend mixtures with disparate core sizes during soft-lithographic confinement. (g) TEM image showing complete selective partitioning of SiO_2_-*g*-PMMA into the less-confined mesa (black-striped) regions. Adapted with permission.^160^ Copyright 2022 American Chemical Society.

ATRP was used to prepare binary or miktoarm polymer brushes on SiO_2_ NPs through multiple synthetic routes, with the “diblock first” strategy yielding phase-separated surface features observable by microscopic analysis.^157^ Alternatively, using an asymmetric difunctional initiator and sequential two-step SI-RDRP, binary mixed homopolymer brushes (P*t*BA/PS)-grafted SiO_2_ NPs were shown to laterally microphase separate into discrete nanodomains (Figure 12d,e).^158^ This spontaneous surface patterning generated multivalent patchy nanoparticles with tunable valency, governed primarily by the average core size relative to the polymer dimensions. These multivalent PGNPs exhibited directional interparticle bonding mediated by nanodomain overlap and displayed environmentally adaptable valency during assembly, arising from competition between brush-driven microphase separation and lattice packing constraints.

Beyond directing discrete particle assembly, interparticle brush interactions in PGNP blends also govern phase separation, miscibility, and confinement-driven morphology evolution in thin films. In binary PS-grafted/PMMA-grafted SiO_2_ PGNP blend films, direct immersion annealing reversibly switched the system between homogeneous and phase-separated states by tuning solvent quality and thereby modulating PGNP phase boundaries and coarsening behavior.^159^ In a related study of imprinted PGNP brush blends, solvent-vapor-annealing soft lithography drove selective partitioning of larger PGNPs into less-confined mesa regions, where segregation arose from the entropic penalty of confinement but was constrained by brush interpenetration and jamming in high-molecular-weight systems (Figure 12f,g).^160^

In more densely packed PGNP solids, interparticle brush interactions further dictate ordered lattice formation and a collective mechanical response. Comb-shaped copolymer brush-grafted SiO_2_ NPs assembled into ordered face-centered-cubic (fcc) lattices, where interparticle spacing was governed by brush architecture as well as steric repulsion and interdigitation between neighboring brushes.^161^ Phase-separated PBA-rich interparticle boundary layers and PMMA-rich domains generated mechanically robust self-standing colloidal crystal films with elastic recovery. Lattice deformation and recovery were controlled by the entropic elasticity of the brush boundary phase and became limited by lattice slippage at higher strain. In the subsequent block copolymer-grafted system, tuning the rubbery/glassy PBA/PMMA ratio similarly modulated particle spacing, structural color, and mechanochromic response, and in situ scattering revealed anisotropic brush deformation together with corresponding changes in interparticle distance under uniaxial and biaxial stretching.^162^

### 4.4 Mechanical Reinforcement

PGNPs offer an effective platform for mechanical reinforcement by coupling nanoscale filler rigidity to tailorable interfacial polymer brush structures. In matrix-free PGNP assemblies, mechanical properties can be regulated through brush-architecture-directed control over interparticle entanglements and deformation pathways. Incorporation of a minor fraction of sufficiently long chains promoted relaxed chain conformations and effective entanglement formation in bimodal brush-grafted systems, thereby enabling robust films with improved fracture toughness at relatively high inorganic content compared with analogous unimodal brush systems (Figure 13a).^163^ Under quasi-static uniaxial deformation, glassy PGNP ultrathin films exhibited apparent crazing behavior and higher strain at break than corresponding homopolymer films of comparable chain length.^164^ Film toughness also depended strongly on grafting density, with intermediate grafting densities outperforming higher ones because increased brush interpenetration promoted more interparticle entanglements per chain. This same structural principle also extended to high-strain-rate impact conditions, where nanoparticle cores acted as nodal cross-links and entangled canopies formed a dual load-sharing network that enhanced viscoplastic deformation and energy dissipation during projectile perforation.^165^

**Figure 13:**
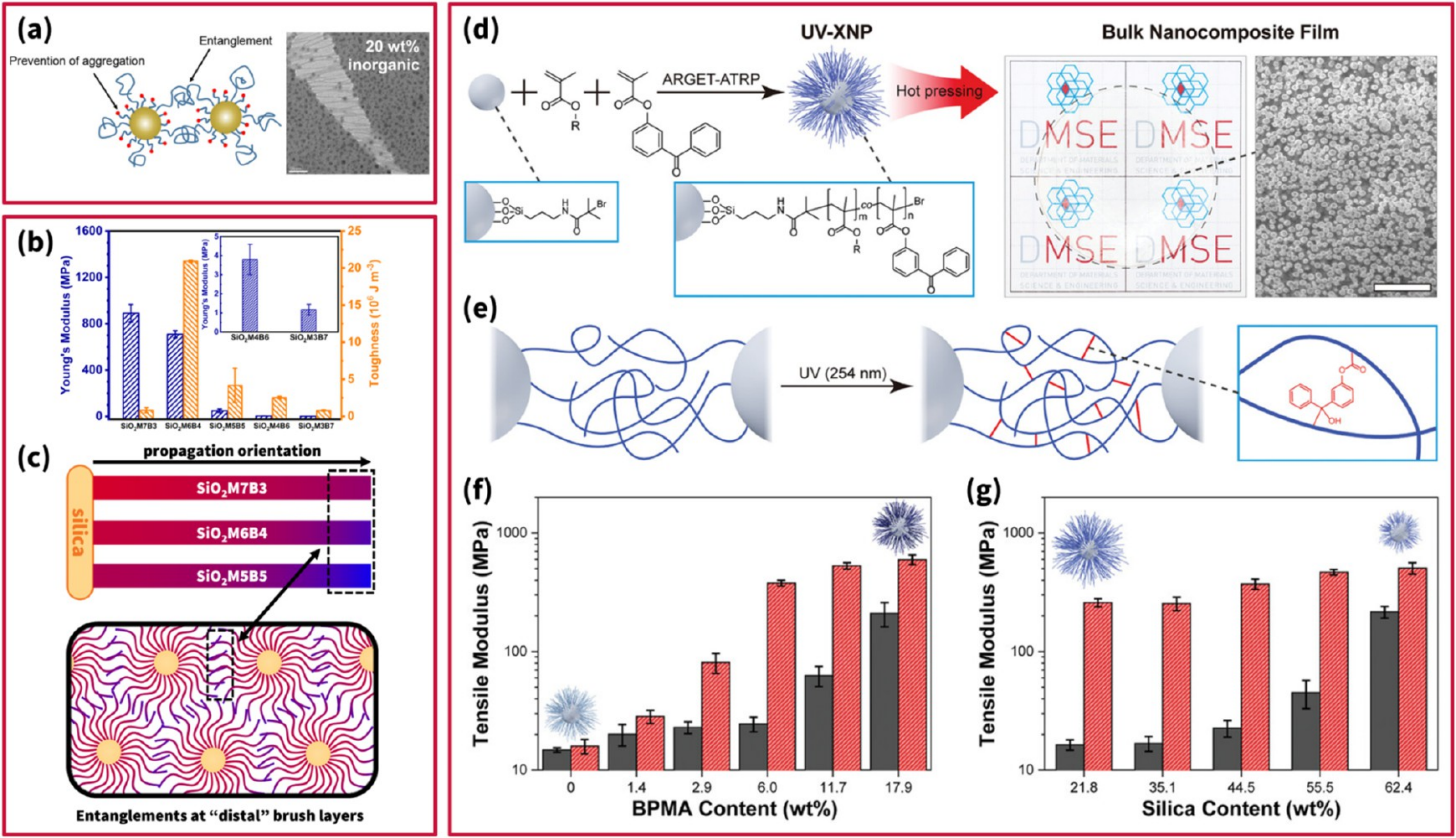
Mechanical reinforcement of PGNPs. (a) Schematic illustration and TEM image showing crack and craze formation in a bimodal brush-grafted nanoparticle system. Scale bar: 100 nm. Adapted with permission.^163^ Copyright 2015 American Chemical Society. (b) Young’s modulus and toughness determined by uniaxial tension testing of spontaneous-gradient copolymer-grafted particle brushes SiO_2_-*g*-PMMA-*grad*-PBA. (c) Proposed origin of the synergistic mechanical behavior of SiO_2_M6B4. Although material stiffness is governed primarily by copolymer composition, toughness arises from interactions between the distal grafted brush layers associated with copolymer propagation orientation. Adapted from ref 139. Copyright 2023 The Authors. Published by American Chemical Society under a CC BY 4.0 license. (d) Synthetic scheme for UV-cross-linkable PGNP composite films containing benzophenone moieties. The optical image shows a demonstrative example of an ≈8 cm bulk nanocomposite film, with an inset SEM image. Scale bar: 500 nm. (e) Schematic illustration of UV-induced cross-linking of grafted brushes via photoinitiation of pendant benzophenone groups. (f) Tensile moduli of different cross-linkable PGNP structures. Black and red bars correspond to UV exposure times of 0 and 24 h, respectively. Adapted from ref 14. Copyright 2025 The Authors. Published by Wiley-VCH GmbH under a CC BY-NC 4.0 license.

Mechanical behavior in PGNP assemblies can be governed by superlattice packing and brush-conformation effects. In self-assembled PS-grafted Au nanocrystal thin films, elastic modulus and hardness were found to depend on film thickness, polymer molecular weight, and the degree of structural disorder, with ultrathin superlattices exhibiting moduli in the GPa range despite their brittle character.^166^ A moderate degree of structural disorder could even increase the elastic modulus due to nonequilibrium brush conformations and residual stress formed during rapid self-assembly.

Chain composition and sequence distribution within grafted copolymer brushes provide an additional handle for tuning the mechanical properties of PGNP assemblies beyond the grafting density or chain length alone. In particular, gradient copolymer brush architectures offered a distinct route to mechanical reinforcement by regulating the segment distribution and the resulting brush microstructure. In SiO_2_-*g*-PBA-*grad*-PMMA films, the reverse-gradient brushes generated topologically induced heterogeneity that was absent in corresponding statistical brush particle systems and linear gradient copolymer analogues, producing cocontinuous PBA-rich and PMMA-rich regions that increased the glass transition temperature, Young’s modulus, and toughness of selected brush particle films.^136^ A subsequent study on SiO_2_-*g*-PMMA-*grad*-PBA with a spontaneous gradient further demonstrated that mechanical performance could be optimized through the combined effects of copolymer composition and propagation orientation (Figure 13b,c).^139^ The SiO_2_ NPs grafted with MMA/BA at a 60/40 molar ratio exhibited the best overall balance of stiffness and toughness, which was attributed to entanglement of the terminal brush layers in the semidilute regime together with an intermediate PMMA fraction in the distal brush region, thereby avoiding the extremes of excessive rigidity or softness.

Covalent interparticle cross-linking reinforces PGNP assemblies by decoupling mechanical robustness from the need for long, highly entangled grafted chains. Poly­(glycidyl methacrylate)-grafted SiO_2_ NPs cross-linked with multifunctional amines formed highly filled composite films that exhibited substantially improved hardness, modulus, scratch resistance, and elastic recovery relative to their non-cross-linked PGNP counterparts.^167^ This concept was further extended to UV-cross-linkable PGNPs containing benzophenone moieties, in which photo-cross-linking transformed soft PGNP films into stiffer solids while enabling spatially resolved stiffness patterning through selective irradiation (Figure 13d–g).^14^ Because this nondisruptive process avoided obvious deformation or discoloration, such hybrid materials further broadened the applicability of cross-linking-based PGNP reinforcement to flexible and optoelectronic systems.

## 5 Structure-Enabled Emerging Applications

### 5.1 3D Printing

Additive manufacturing, commonly known as 3D printing, is a layer-by-layer fabrication approach that enables rapid and customizable production of complex geometries that are difficult to achieve using conventional subtractive or molding-based manufacturing methods.^168,169^ PGNPs have emerged as versatile functional additives for 3D printing processes. By combining nanoparticle cores with tunable polymer brush coronas, PGNPs provide precise control over interfacial interactions and dispersion within printable matrices. As a result, PGNPs can tailor the rheological properties of printing formulations, enabling controlled viscosity and flow behavior that support dimensionally stable and high-resolution printing, while simultaneously enhancing the mechanical and functional properties of the resulting printed composites.^170^

SiO_2_-*g*-PMMA illustrated how PGNP-mediated interfacial engineering could improve the performance of parts produced by fused filament fabrication (FFF), where the quality of interactions between deposited filaments strongly governed the final mechanical properties.^171^ When incorporated into a PMMA matrix, the grafted nanoparticles formed localized clustered nanostructures and increased the melt storage modulus and complex viscosity, indicating enhanced interdiffusion and entanglement between the grafted and matrix chains. These nanoscale effects translated into improved properties in the printed parts, where Young’s modulus, tensile modulus, and ultimate tensile strength increased substantially relative to unfilled PMMA. In contrast to simple blending of bare SiO_2_, the grafted nanoparticles provided more efficient reinforcement at lower loading, highlighting PGNP interfacial engineering as an effective route to improved bead-to-bead adhesion and thermomechanical performance in FFF-printed polymers.

Random copolymer-grafted SiO_2_ NPs, bearing poly­(methyl methacrylate-*random*-2-uriedo-[1H]-pyrimidinone methacrylate) [P­(MMA-*r*-UPyMA)] grafts, represented an effective additive platform for FFF-printed PMMA.^172^ The UPy-containing graft corona introduced reversible, multipoint hydrogen-bonding interactions that enhanced bead-to-bead adhesion, while the SiO_2_ core contributed structural reinforcement. In combination with graft-matrix entanglements, these interactions improved additive dispersion, reduced interbead voids, and promoted more effective welding of printed filaments, especially when the nanocomposites are prepared by solution casting prior to extrusion. As a result, the printed parts showed improved thermomechanical performance relative to unfilled PMMA and to analogous materials prepared by simple mechanical mixing.

Dynamic covalent bond exchange offered an effective route to self-healing in 3D-printable elastomeric photoresins.^173^ Thermally activated retro-thiol-Michael reactions enabled network rearrangement upon heating, while poly­(2-hydroxyethyl acrylate) (PHEA)-grafted SiO_2_ NPs served as multivalent reinforcing fillers. Through brush–matrix entanglement and hydrogen bonding, the PGNPs enhanced stiffness and strength without significantly reducing the polymer mobility required for efficient healing, thereby helping to overcome the usual trade-off between mechanical robustness and self-healable capability in printable elastomers (Figure 14).

**Figure 14:**
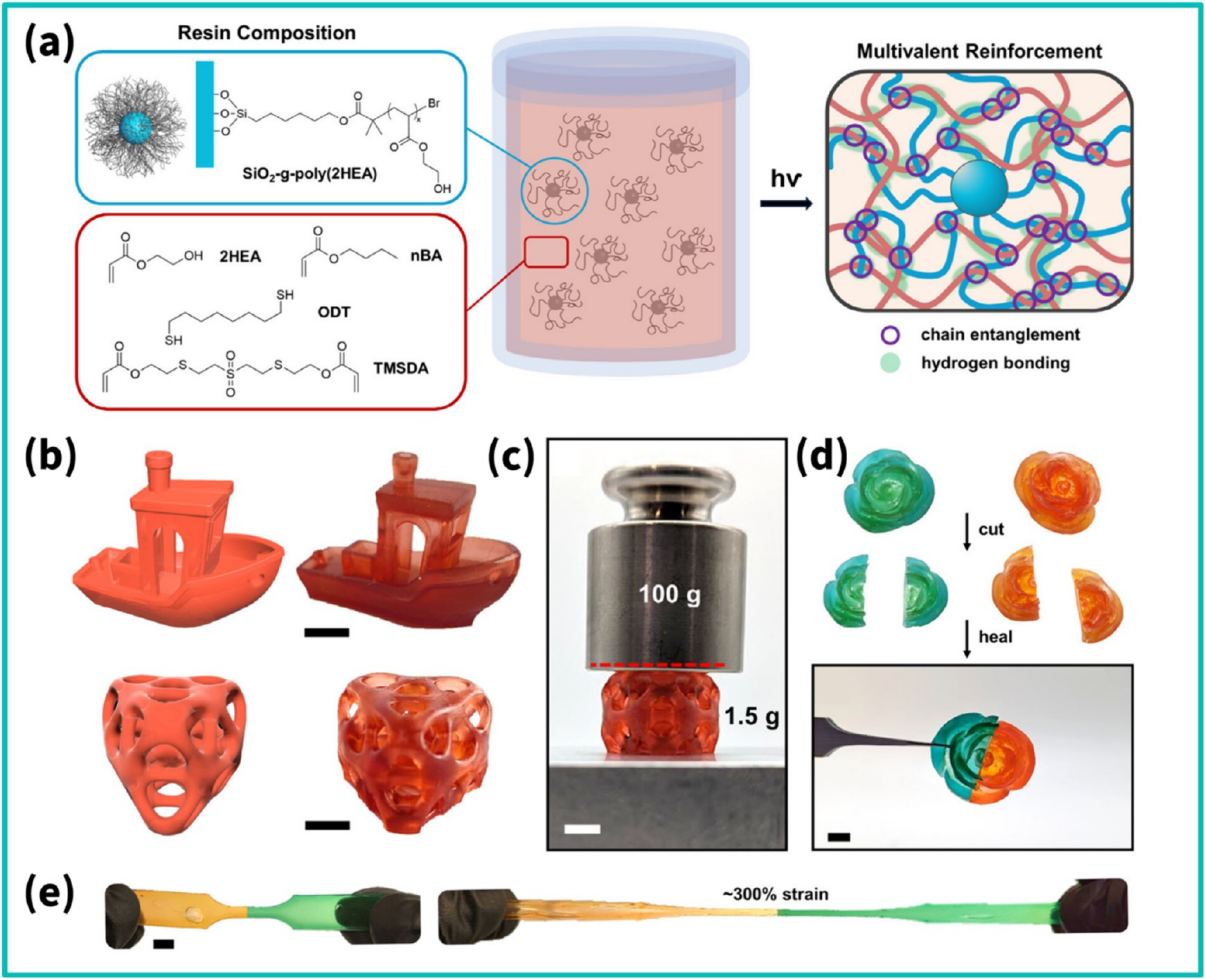
Multivalent reinforcement of 3D-printable self-healing elastomers using PGNPs. (a) Schematic illustration of the resin formulation and conceptual multivalent reinforcement from PGNP incorporation. (b) Complex 3D-printed objects with 4 wt % PGNPs. (c) Load-bearing capability under compression (original height marked by dotted red line). (d) Cut-and-heal behavior. (e) High extensibility of the printed elastomers after healing. Scale bars: 5 mm. Adapted with permission.^173^ Copyright 2024 American Chemical Society.

### 5.2 Self-Healing and Adaptive Materials

Self-healing refers to a material’s ability to autonomously restore mechanical integrity after damage under appropriate conditions or stimuli without sacrificing its original performance. Advances in self-healing polymers have significantly enhanced structural durability, extending service lifetime and improving resource efficiency.^174,175^ Among the various mechanisms enabling self-healing, hydrogen bonding represents a widely employed driving force. These interactions can also be incorporated into PGNP systems to generate dynamic and recoverable nanoparticle assemblies. For instance, amide-bearing polyacrylates-grafted SiO_2_ NPs established reversible hydrogen bonding between neighboring brushes, forming dynamic interparticle networks that yielded mechanically robust nanocomposites capable of thermally activated self-healing.^176^

More recently, attention has shifted to self-healing driven by weaker intermolecular interactions, particularly van der Waals (vdW) forces, in conventional commodity polymers. This phenomenon was first demonstrated in approximately equimolar poly­(methyl methacrylate/*n*-butyl acrylate) [P­(MMA/BA)] copolymers synthesized via free radical polymerization or ATRP.^177^ These materials exhibited ambient mechanical recovery, which was attributed to a proposed “key-and-lock” interdigitation of alternating MMA/BA motifs. Computational analyses suggested that this microstructural arrangement increased the cohesive energy density between chains, facilitating the reversible restoration of intermolecular vdW interactions after damage. Self-healing in commodity polymers emerged simply from appropriate chain conformation and copolymer microstructure, without requiring complex molecular design.^178^

Building on this premise, the role of copolymer sequence was first examined through Monte Carlo simulations^179^ and subsequently decoupled experimentally by synthesizing nearly equimolar P­(MMA/BA) with controlled alternating, statistical, and gradient architectures.^180^ Although alternating copolymers possessed the highest MMA/BA dyad frequency, their faster recovery was attributed to microstructural uniformity rather than dyad abundance (Figure 15a,b).^181^ Notably, despite exhibiting the most rapid healing, these alternating systems displayed comparatively weaker mechanical properties. In contrast, gradient systems showed enhanced mechanical strength at the expense of slower repair. Small-angle neutron scattering (SANS) revealed subtle glassy MMA-rich clusters that likely pinned polymer chains and retarded the relaxation dynamics. Extending this analysis to a broader range of acrylate-based copolymers, and even to PMA homopolymers, further demonstrated that recovery kinetics primarily scaled with bulk viscoelastic properties and chain mobility.^182^ These findings indicated that specific key-and-lock interactions are not a prerequisite for mechanical recovery; instead, they are governed by (co)­polymer glass transition temperature and relaxation dynamics.

**Figure 15:**
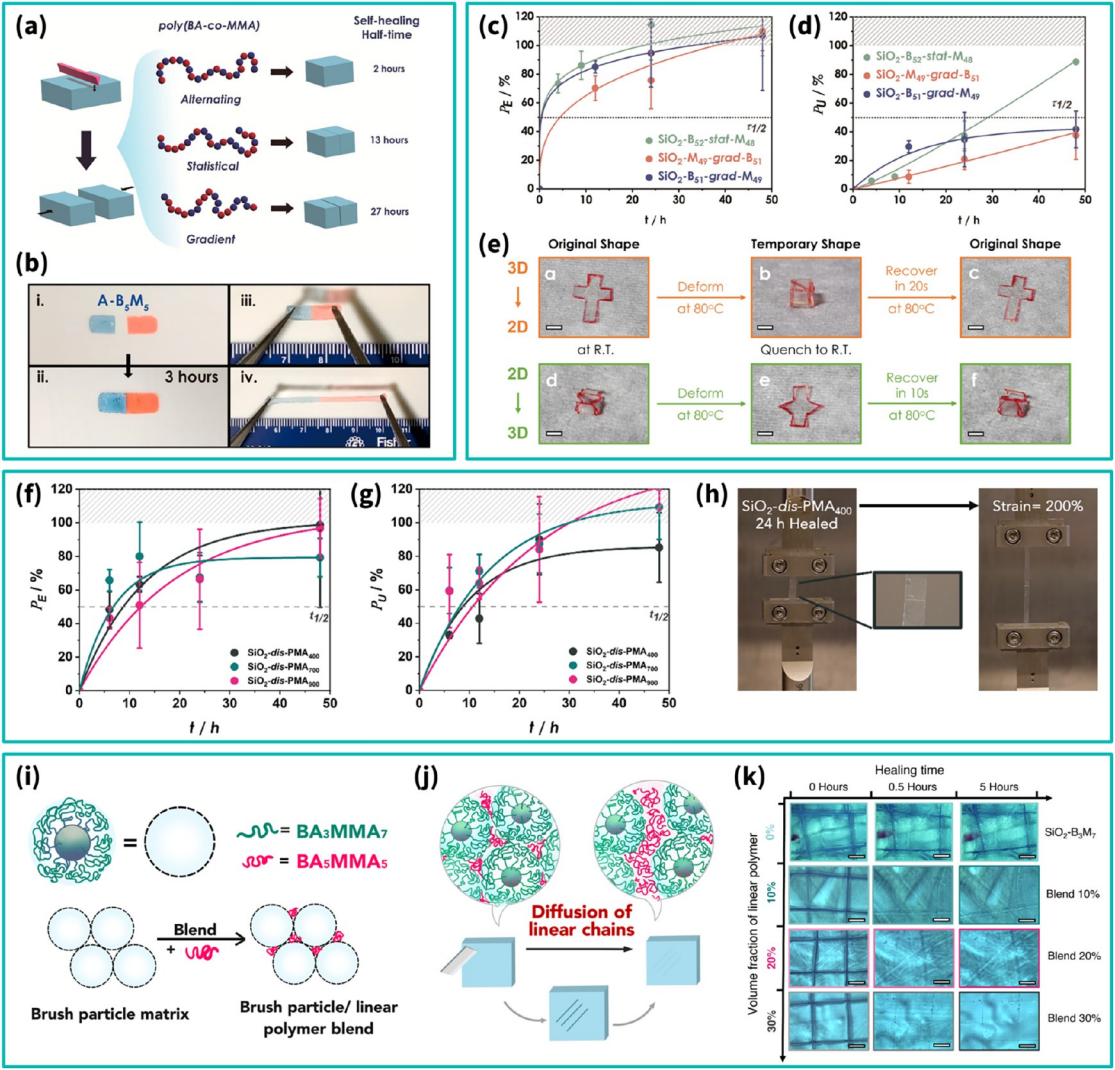
Self-healing behavior in acrylate-based PGNP systems. (a) Schematic illustration of sequence-dependent self-healing in P­(MMA/BA) copolymers. (b) Alternating P­(MMA/BA) bulk film after healing showing ∼350% extensibility 3 h after rejoining. Adapted from ref 181. Copyright 2023 The Authors. Published by American Chemical Society under a CC BY 4.0 license. Recovery of (c) Young’s modulus (*P*_E_) and (d) toughness (*P*_U_) of self-healed SiO_2_-*g*-P­(MMA/BA) films after rejoining at 50 °C followed by annealing at 100 °C. Subscripts denote copolymer compositions; *stat* and *grad* indicate statistical and gradient architectures, respectively. (e) Shape-memory behavior of SiO_2_-*g*-BA_45_-*stat*-MMA_55_. Adapted from ref 137. Copyright 2023 The Authors. Published by American Chemical Society under a CC BY 4.0 license. Recovery of (f) Young’s modulus (*P*_E_) and (g) toughness (*P*_U_) of high-dispersity SiO_2_-*g*-PMA films. (h) Healed high-dispersity SiO_2_-*g*-PMA film demonstrating ∼ 200% extensibility after rejoining and annealing for 24 h. Adapted from ref 183. Copyright 2025 The Authors. Published by American Chemical Society under a CC BY 4.0 license. (i) Schematic illustration of integrated self-healing in blends of statistical P­(MMA/BA) linear copolymer and PGNPs. Subscripts denote copolymer compositions. (j) Proposed healing mechanism in the blended system via diffusion of self-healing linear copolymer through interstitial regions to damage sites. (k) Stereomicroscopic images of scratch healing (performed at 70 °C) for blends containing different volume fractions of linear BA_5_MMA_5_ copolymer. Scale bars: 500 μm. Adapted from ref 184. Copyright 2025 The Authors. Published by American Chemical Society under a CC BY 4.0 license.

Considering the limited mobility of nanoparticle cores within bulk matrices, acrylate-based (co)­polymer-grafted nanoparticles represent promising candidates for self-healable hybrid materials. The densely tethered polymer brush architecture suppressed viscous flow and slowed cooperative relaxation compared to their unattached free counterparts during the healing process.^137^ By tailoring the copolymer composition and sequence, copolymer brush-grafted particle films could uniquely integrate self-healing with shape-memory behavior. Statistical copolymer sequences promoted faster recovery of modulus and toughness (Figure 15c–e), while increased MMA content significantly enhanced stiffness without compromising healing efficiency. These brush particle hybrids could store and release strain energy to restore programmed shapes, enabling a “recall-and-repair” mechanism.

Increasing the dispersity of grafted polymer brushes significantly enhanced the toughness, strain-to-fracture, retardation time, and rate of toughness recovery in brush particle solids, while leaving the glass transition temperature and Young’s modulus largely unchanged.^183^ High-dispersity systems recovered more rapidly because broader dynamic heterogeneity and a small fraction of high-molecular-weight chains promoted more effective entanglement network reformation and brush interdigitation (Figure 15f–h). In another work, blending self-healable copolymer brush particles (*d*_core_ ≈ 110 nm) with unattached free P­(MMA/BA) copolymer enabled glassy materials that combined high modulus (∼1 GPa), structural color, and low-temperature healing via confinement-driven segregation (Figure 15i–k).^184^ The linear copolymer selectively localized within the interstitial regions of a rigid, disordered hyperuniform brush particle template, where it diffused into damaged zones to restore structure while preserving the template’s mechanical framework and optical order. The synergistic coupling of a mechanically jammed brush particle network with a mobile, intrinsically self-healing filler produced reprocessable, shape-memory- and damage-resistant materials that transcended the limitations of conventional intrinsic or extrinsic healing strategies.

### 5.3 Gas Separation

Gas separation involves using selective membranes or materials to differentiate gas species based on differences in size or diffusivity, enabling efficient purification or mixture separation.^185,186^ PGNPs are attractive for gas-separation membranes, because the grafted brush layer can simultaneously improve nanoparticle dispersion and interfacial compatibility while tuning local free volume, chain packing, and gas sorption characteristics. As a result, PGNP-based membranes can enhance CO_2_ permeability, maintain or improve gas selectivity, and provide better mechanical robustness than conventional mixed-matrix or neat polymer membranes.

PMA- or PMMA-grafted SiO_2_ NP membranes behaved as spatially heterogeneous transport media, in which distinct free-volume pathways could be selectively manipulated through the addition of free polymer chains (Figure 16a,b).^125^ When free chains with molecular weights comparable to those of the grafts segregated into distal interstitial regions between neighboring nanoparticles, they preferentially suppressed the diffusion of larger gas molecules, enabling up to 2 orders of magnitude increases in gas selectivity while only moderately reducing permeability. Subsequent work showed that in densely grafted PGNP assemblies, gas permeability was governed by brush extension and nanoparticle volume fraction, reaching a maximum near an apparently universal loading where the packing-induced chain extension free energy was the highest.^187^ Whereas small gas molecules retained activation energies close to those in the neat polymer and therefore benefitted mainly from faster local chain dynamics in the brush layer, larger gas molecules exhibited reduced activation energies due to preferential sorption and transport through lower-density interstitial regions in the nanoparticle assembly.^188^

**Figure 16:**
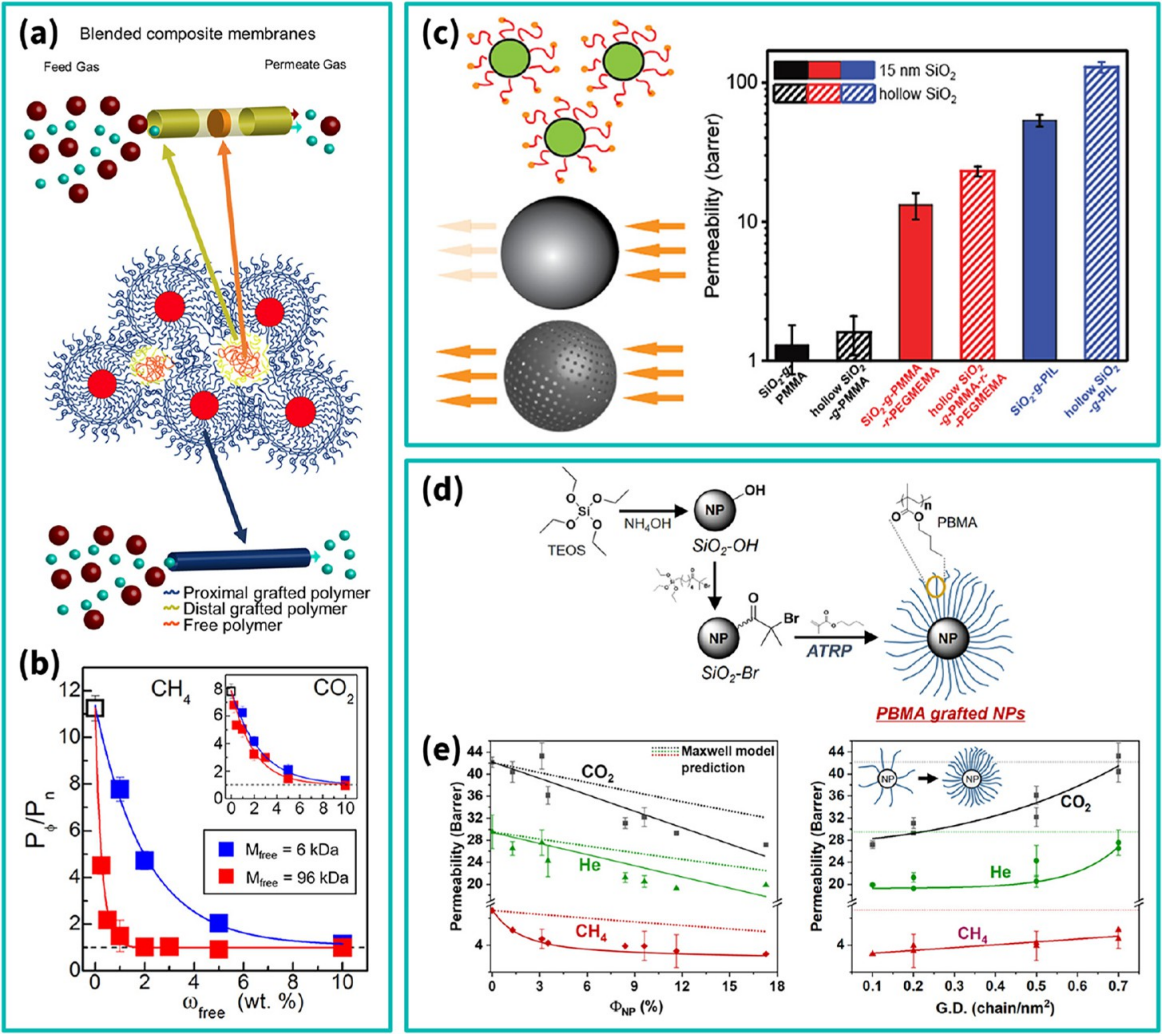
Representative PGNP-enabled strategies for gas separation membranes. (a) Schematic illustration of gas transport in the blended PGNP nanocomposites. Free polymer chains with molecular weights comparable to those of the grafts preferentially segregate into distal brush regions and more strongly suppress transport of larger gases, whereas smaller gases are transported more readily through the grafted polymer phase. (b) CH_4_ permeability enhancement and CO_2_ permeability (inset) in PMA-grafted PGNPs as a function of the added free-chain content. Adapted with permission.^125^ Copyright 2020 American Chemical Society. (c) CO_2_ permeability of membranes based on polymer-grafted solid and hollow SiO_2_ NPs. Adapted with permission.^189^ Copyright 2021 American Chemical Society. (d) Synthetic route to PBMA-grafted SiO_2_ NPs. (e) Gas permeability of SiO_2_-*g*-PBMA membranes as a function of SiO_2_ volume fraction and grafting density. Adapted with permission.^191^ Copyright 2021 American Chemical Society.

Both the core structure and brush architecture can be leveraged to improve the CO_2_ separation performance while preserving mechanical robustness. Compared with nonionic PMMA- and PMMA-*random*-POEOMA-grafted analogues, poly­(ionic liquid) (PIL)-grafted SiO_2_ NPs exhibited much higher CO_2_ permeability.^189^ Additionally, replacing solid SiO_2_ with hollow porous SiO_2_ further enhanced the permeability by introducing internal transport pathways through the core (Figure 16c). Related work showed that SiO_2_-*g*-PMMA-*block*-PIL retained nearly the same CO_2_ permeability and CO_2_/N_2_ selectivity as SiO_2_-*g*-PIL, but delivered improved Young’s modulus because the PMMA block provided additional structural support without sacrificing the PIL-enabled transport characteristics.^190^

Poly­(butyl methacrylate) (PBMA)-grafted SiO_2_ NP membranes showed that gas transport could be tuned through grafting density and nanoparticle loading.^191^ Increasing inorganic fraction generally decreased gas permeability because the particles acted as diffusion barriers, whereas higher grafting density promoted more favorable microstructures that preserved CO_2_ and He transport while more strongly suppressing CH_4_ permeation (Figure 16d,e). These effects arose from changes in particle organization and local free-volume structure, together with favorable interfacial CO_2_ sorption, collectively improving gas discrimination relative to unattached free PBMA. Nanoparticle dispersion also governed PGNP membrane performance.^192^ Well-dispersed PMA-grafted SiO_2_ NPs yielded higher CO_2_ permeabilities while preserving CO_2_/CH_4_ selectivity relative to agglomerated counterparts, as even slight aggregation increased the particle size and suppressed permeability gains.

### 5.4 Energy Storage and Battery Interfaces

Rechargeable batteries are indispensable for modern technologies ranging from portable electronics and electric vehicles to large-scale energy storage, creating an urgent demand for materials that improve safety, capacity, and cycling performance.^193–197^ PGNPs have recently emerged as a versatile platform for battery systems because their hybrid architecture combines the functional properties of inorganic nanocores with the tunable interfacial characteristics of polymer brushes.^198–201^ By tailoring brush composition, grafting density, and interparticle interactions, PGNPs can enhance ionic conductivity, regulate electrode–electrolyte interphases, and suppress dendrite formation, offering promising strategies for next-generation battery materials.^202–204^

PGNPs have been explored as functional additives in advanced electrolyte membrane systems for battery-related applications.^205^ For example, Fe_3_O_4_ nanoparticles grafted with PMMA-*block*-polystyrenesulfonate (PSS) demonstrated that the brush architecture strongly influenced nanoparticle dispersion, ion-pair dissociation, and ionic conductivity in ionic liquid/cosolvent media.^206^ In these systems, PMMA preferentially interacted with the anions, whereas PSS associated with the cations of the ionic liquid, thereby modulating the distribution of free mobile ions within the hybrid electrolyte. PGNPs bearing longer PMMA chains and lower sulfonation levels exhibited the highest conductivity, particularly in polar solvent mixtures, where improved solvation promoted greater ion mobility. In a subsequent study, this concept was extended to single-ion conduction using PIL-grafted Fe_3_O_4_ nanoparticles (Figure 17a,b).^207^ Compared with the neat PIL system, Fe_3_O_4_-*g*-PIL exhibited enhanced molar conductivity and reduced barriers for ion transport, which could be attributed to confinement of anions within interparticle regions and ladder-like ion hopping along connected polycation brushes. The observed conductivity enhancement depended on the underlying particle network, highlighting particle percolation and graft connectivity as key design parameters for single-ion-conducting electrolytes.

**Figure 17:**
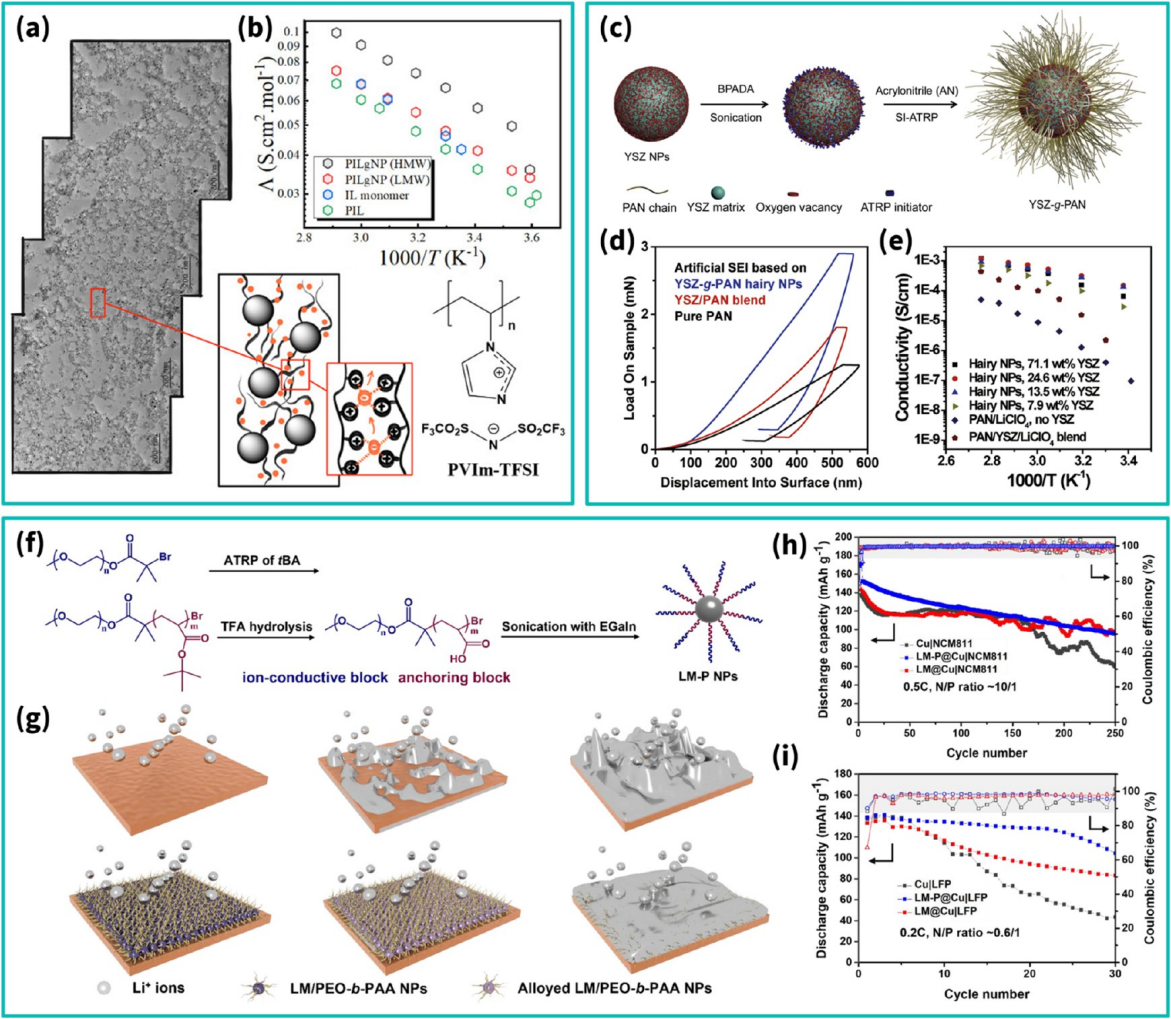
Representative PGNP-enabled strategies in battery-related systems. (a) Fe_3_O_4_-*g*-PIL exhibiting single-ion conduction. (b) Molar conductivities of Fe_3_O_4_-*g*-PIL with higher-molecular-weight (HMW) and lower-molecular-weight (LMW) grafts, together with neat PIL and ionic liquid monomer. Adapted with permission.^207^ Copyright 2024 American Chemical Society. (c) Synthetic scheme for YSZ-*g*-PAN. (d) Load–displacement curves of artificial SEI based on YSZ-*g*-PAN, noncovalent YSZ/PAN blends, and pure PAN. (e) Arrhenius plots of the ionic conductivity of artificial SEI prepared from LiClO_4_ mixed with YSZ-*g*-PAN of different inorganic fractions, a YSZ/PAN blend, or pure PAN. Adapted from ref 209 with permission. Copyright 2020 The Authors. Published by Elsevier Ltd. (f) Synthetic route to PEO-*block*-PAA copolymer-stabilized EGaIn nanoparticles (LM-P). (g) Schematic illustration of Li nucleation and deposition behavior on bare Cu and LM-P@Cu. Capacity retention as a function of cycle number for (h) Li||NCM811 cells at 0.5C and (i) Li||LFP cells at 0.2C, with Li predeposited on bare Cu, LM@Cu, and LM-P@Cu substrates. The first three cycles are formation cycles at 0.1C. Adapted with permission.^214^ Copyright 2022 American Chemical Society.

PGNPs have also been investigated for constructing artificial solid electrolyte interphases (SEIs) on lithium metal anodes because they combine the mechanical robustness of inorganic nanoparticles with the conformability and interfacial adaptability of polymeric coatings.^208^ In one example, PAN chains were grafted from oxygen-vacancy-rich yttria-stabilized zirconia (YSZ) nanoparticles via SI-ATRP to produce PGNPs that could be solution-cast into uniform protective layers on lithium metal (Figure 17c–e).^209^ The resulting hybrid SEI exhibited high ionic conductivity (∼10^–4^ S·cm^–1^) together with improved mechanical robustness, while promoting homogeneous Li^+^ flux at the electrode interphase and effectively suppressing lithium dendrite formation. Consequently, lithium metal cells protected by these PGNP-based coatings demonstrated long-term cycling stability (>2500 h) with low overpotential, as well as improved capacity retention in full cell configurations. Similarly, elastic hairy SiO_2_ nanoparticles grafting poly­(oligo­(ethylene glycol) methyl ether methacrylate) (POEOMA_500_, average *M*_n_ = 500) via SI-ATRP were applied as separator coatings or artificial SEIs, where the highly entangled polymer brush layer enabled uniform lithium deposition and improved cycling stability with ∼86% capacity retention after 500 cycles.^210^

Another example based on POEOMA_500_-grafted SiO_2_ NPs demonstrated that tuning grafting density could regulate ionic transport through the brush layer and significantly extend cycle life.^211^ Optimized PGNP coatings with an intermediate grafting density (∼0.46 chains·nm^–2^) promoted dense and uniform lithium deposition, extending the cycle life of symmetric cells to ∼275 cycles compared with bare lithium. Mechanistic analysis indicated the improved performance arose primarily from regulated ionic transport and more homogeneous plating/stripping behavior, rather than from the mechanical stiffness of the coating. Subsequent work further examined how particle brush architecture influenced lithium deposition, showing that grafting density, inorganic content, and brush molecular weight together determined the balance between ionic conductivity and mechanical protection.^212^ In particular, high-molecular-weight brushes could enter semidilute brush regimes in which chain entanglements enhanced resistance to dendrite growth but excessive molecular weight also increased polarization. Meanwhile, grafting density strongly governed ion transport: excessively high densities hindered Li^+^ mobility by reducing free volume and increasing transport tortuosity, whereas overly low densities provided insufficient polymer content for effective ion dissociation. As a result, intermediate grafting density and molecular weight provided the most favorable overall battery performance.

Beyond metal oxide and SiO_2_-based systems, EGaIn liquid metal nanoparticles were explored as deformable battery components because of their lithiophilicity and capability of regulating Li nucleation and deposition.^213^ EGaIn nanoparticles stabilized by poly­(ethylene oxide)-*block*-poly­(acrylic acid) (PEO-*b*-PAA) copolymers were developed as a scalable spray-coated modification layer on Cu current collectors (Figure 17f–i).^214^ The resulting polymer-stabilized EGaIn PGNPs promoted homogeneous Li deposition and suppressed dendritic growth. Consequently, the modified current collector enabled stable cycling of 30 μm Li anodes in Li||NCM811 full cells while also supported ultrathin 1 μm Li anodes in Li||LiFePO_4_ cells under conditions approaching anode-free configurations. A related approach introduced copolymer-grafted EGaIn nanoparticles in which ionomer-containing polymer binders suppressed nanoparticle reaggregation while providing ionic transport channels that facilitated Li^+^ migration to the liquid metal surface.^215^ As a result, these hybrid EGaIn-polymer electrodes exhibited high specific capacity, together with improved cycling stability (∼85% capacity retention after 500 cycles).

## 6 Conclusions and Future Perspectives

PGNPs have emerged as a versatile class of all-in-one hybrid nanomaterials, in which nanoparticle cores and polymer coronas are integrated into structurally programmable building blocks. Continued advances (especially in the past decade) were discussed in nanoparticle surface functionalization, surface-initiated polymerization, and architectural regulation, together with precise control over grafting density, composition, and dispersity of the brush layers. These synthetic developments have established clear connections between nanoscale brush structure and collective properties, including conformation, interparticle interactions, assembly, and mechanical response. Consequently, PGNPs have evolved beyond model colloidal systems or compatibilized fillers into a broad materials platform that bridges polymer chemistry, colloid science, and nanomaterials engineering. Recent progress further demonstrates that PGNPs are being translated into increasingly diverse application spaces, including 3D printing, self-healing materials, gas separation membranes, and battery-related systems. Across these fields, material performance is dictated not only by the chemical identity of the core and grafted polymer but also by the hierarchical organization and interfacial characteristics. This structure-enabled design principle distinguishes PGNPs from conventional nanocomposites fabricated by blending and highlights the importance of the polymer architecture as a central handle for tuning macroscopic function.

Looking forward, continued integration of precision polymer synthesis, advanced characterization, predictive modeling, and application-oriented materials design is expected to further expand the scope of PGNPs. Extension of these concepts to porous, deformable, organic, hybrid, electronically active, and optically responsive nanoparticle platforms, together with loop, cylindrical, and hyperbranched brush architectures, should open new opportunities in photonics, soft electronics, separations, catalysis, and energy-related materials. Overall, PGNPs provide a powerful framework for translating molecular-level control of interfacial polymer architecture into emergent macroscopic performance, and the field is well positioned to play an increasingly important role in next-generation hybrid polymer materials.
